# Phenoxazine Radical as a Positive Material for Neutral pH Aqueous Flow Batteries

**DOI:** 10.1021/acsaem.5c00225

**Published:** 2025-05-05

**Authors:** Eduardo Martínez-González, Ali Tuna, Pekka Peljo

**Affiliations:** † Department of Mechanical and Materials Engineering, Research Group of Battery Materials and Technologies, 98743 University of Turku, Vesilinnantie 5, Turku FI-20014, Finland; ‡ Department of Chemistry, 8058 University of Turku, Henrikinkatu 2, Turku FI-20014, Finland

**Keywords:** neutral pH flow batteries, aqueous-soluble phenoxazine, stable radical, *posolyte*, mixed mechanism, diffusion-controlled, adsorption interactions

## Abstract

Understanding electron transfer reactions in phenoxazine aqueous-soluble electroactive materials is crucial for developing flow battery (FB) electrolytes, especially for the positive side. Here, we prepared a water-soluble phenoxazine methyl celestine blue compound (**mCB**) to demonstrate its relatively high redox potential and study its reversible redox chemistry in aqueous KCl solutions. This flow battery (FB) electrolyte exhibited full capacity retention when tested in a symmetrical cell operated at 86% capacity during 55 charge–discharge cycles. The stability of the radical species formed during the one-electron reduction process of **mCB** (to obtain the positive electrolyte of the FB cell) was also characterized by cyclic voltammetry and electron paramagnetic resonance (EPR) spectroscopy. This electrolyte was also tested against a viologen-based *negolyte*, and the detected capacity loss (after 55 cycles) was related to a degradation mechanism of the **mCB** compound undergoing proton oxidation reactions. The experimental results suggest that a more exhaustive characterization by cyclic voltammetry be considered when analyzing FB electrolytes, in order to also take into account the possible effect of inner-sphere electron transfer reactions on the reaction mechanism and its electrochemical parameters.

## Graphical abstract

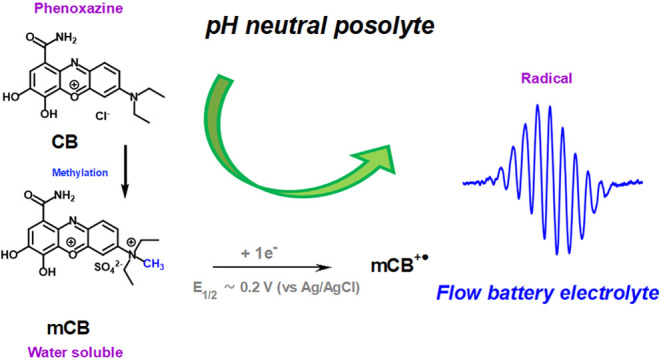

## Introduction

Flow batteries (FBs) are stationary energy storage devices that could become vital for transitioning toward a sustainable energy model supplied by intermittent sources, such as sun and wind.^1–3^ This technology stores electricity in *posolyte* and *negolyte* electrolytes contained in two separate external tanks. As the amount of stored electricity depends on the tank volume, FBs are one possibility for enabling large-scale and long-duration electricity storage. As a main advantage over enclosed technologies (such as Li-ion batteries), FBs can be scaled separately in terms of power and energy by changing the electrode area and tank size, respectively. In FBs, the electrolytes are pumped into different compartments of an electrochemical cell, where reversible oxidation and reduction reactions at electrodes are fundamental processes to charge–discharge the redox active electrolytes of the device.^1^ *Negolyte* materials are characterized by a relatively low reduction potential value, while the reverse is true for the oxidation process of *posolytes*.

Commercially available flow batteries require vanadium, classified as a critical raw material by EU. The relatively high cost of the vanadium electrolyte impedes a broader commercialization of the vanadium-based technology.^4^ Therefore, in recent years, efforts are intensifying to develop systems operating with water-soluble organic electroactive compounds,^1–3^ which often exhibit faster electrode kinetics than inorganic compounds, are abundant in nature, and have tunable chemical (e.g., solubility) and electrochemical (e.g., redox potentials) properties. A wide variety of aqueous *Negolyte* materials for storing 1 or 2 electrons per electroactive molecule have been identified.^5–9^ Designing *posolyte* electrolytes continues to be a challenge mainly because their electrogenerated species (formed upon charging the cell) are unstable in aqueous media.^10^

There are scarse *posolyte* solutions discovered so far, among which solutions composed of organometallic electroactive-molecules have shown better performance.^1,5,11,12^ Some of these systems can evolve into stable charged species (upon charging the cell) at different pH values and in some cases, also in the presence of oxygen.^12^ So, replacing these materials with organic electroactive compounds represents a serious problem for two main reasons. The first one is related to the low redox potential of most compounds in water solvents, and the second one to the faradaic imbalance and/or degradation of the FB electrolytes by the infiltration of small amounts of oxygen into the tanks.^10,13,14^

In the context of *posolytes*, the following water-soluble organic electroactive compounds have demonstrated successful performance: under acidic conditions, methylene blue^9,13,15^ and some quinone compound derivatives (including hydroxyquinone and quinone sulfonic acid structures) were tested as two-electron transfer systems,^16,17^ while a spirobifluorene-based compound and pyrene-4,5,9,10-tetraone-1-sulfonate were tested as four-electron transfer species;^18,19^ TEMPO radicals,^20,21^ and a water-soluble phenazine radical cation^22^ have been studied in neutral media; to the best of our knowledge, there is no completely organic molecule that can successfully be charged-discharged at pH 14, which is a typical condition for testing alkaline flow batteries. Therefore, the improvement of aqueous organic FBs is dependent not only on finding new organic *posolytes* (from which stable charged species can be electrogenerated) but also on designing structures capable of undergoing reversible electron transfer reactions in the presence of some traces of oxygen.

An interesting based-structure that was recently introduced as an electroactive material for aqueous organic FBs is phenoxazine;^1,10,23^ its derivatives are also used in medical and electronic applications, such as dye-sensitizers of solar cells, spintronics, light-emitting diodes, redox shuttle, and battery cathode materials.^1,24–26^ Considering that substituent groups OH and COOH provide solubility at alkaline conditions, Martínez et al.,^1^ proposed the use of commercially available gallocyanine compound as a two-electron storage *negolyte* for alkaline flow batteries, reporting a good cycling stability for the material. The authors did not use a glovebox for the experimentation; they bubbled nitrogen into the tanks. Likewise, these phenoxazine molecules present a relatively high redox potential, as they have positive charges in their structure. Taking advantage of this, compound basic blue 3 was successfully tested as a two-electron storage *posolyte* in 3.5 mol L^–1^ H_2_SO_4_.^23^

Finally, phenoxazine-based skeleton having a tetraalkylammonium moiety **–NR**_**4**_^**+**^ was recently tested as a *posolyte* material in neutral aqueous FBs,^10,27^ but the cell lost its storage capacity in the first charge–discharge cycles. To avoid the mechanism of decomposition of this molecule and for future work, the authors suggested the incorporation of more substituent groups into its structure. Therefore, the feasibility of using aqueous neutral phenoxazine solutions as *posolyte* FB electrolytes continues to be under investigation.

To improve the development of phenoxazine-based batteries, we took commercially available compounds gallocyanine **GAL** and celestine blue **CB** as phenoxazine model systems to study their reduction mechanism under neutral and alkaline conditions. A compound having two positive charges in its structure (methyl celestine blue, **mCB**, Figure 1) and being more soluble (in neutral and alkaline aqueous media) than **GAL** and **CB** compounds was obtained by methylating **CB**. The charge–discharge cycling stability of **mCB**-KOH and **mCB**-KCl solutions was tested in symmetric cells, demonstrating reversible reduction processes involving two and one electrons per electroactive molecule, respectively. The system **mCB**-KCl exhibited a relatively high redox potential (more than 0.2 V vs Ag/AgCl, at 0.04 mol L^–1^ KCl) and the cell operated with 86% capacity without losing it during 55 charge–discharge cell cycles. The radical species formed upon charging this *posolyte* solution was characterized by cyclic voltammetry and electron paramagnetic resonance (EPR).

**Figure 1:**
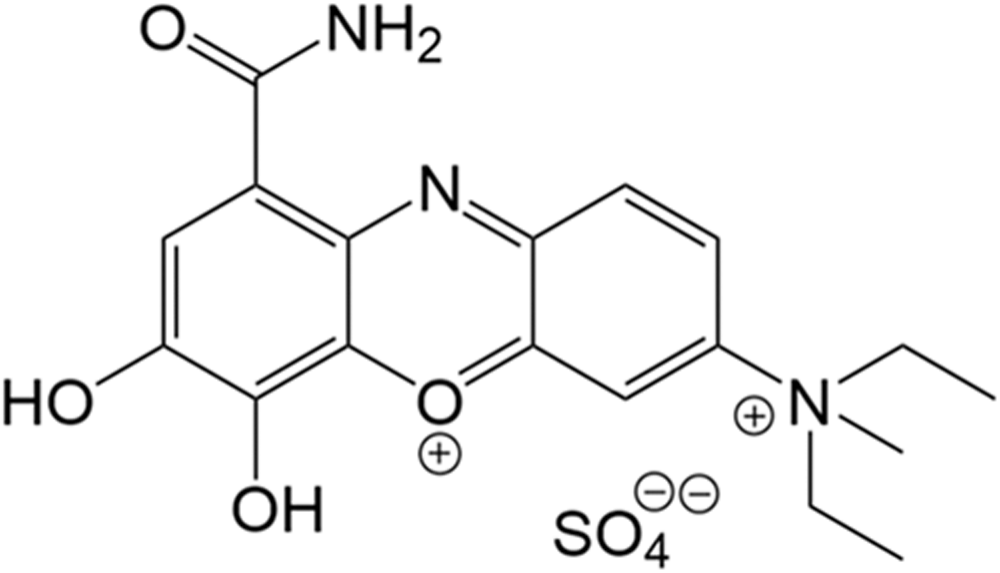
Chemical structure of the studied methyl celestine blue compound **mCB**.

Finally, 55 charge–discharge cycles of a 0.73 V battery cell were tested by pairing system **mCB**-KCl to a *negolyte* tank containing methyl viologen in KCl. The capacity loss detected in the latter system was related to a degradation mechanism involving proton oxidation at the **mCB** electroactive compound. Since conventional methods for obtaining diffusion coefficients and electron transfer rate constant values are not applicable in this case, the kinetic and thermodynamic parameters controlling the overall mixed mechanism composed of adsorption interactions and diffusion-controlled reactions, at the modified electrode surface, were estimated by voltammetric simulations.

## Methods

### Chemicals

Electrochemical experiments were carried out for electroactive compounds **GAL** (99%), **CB** (99%), 1,1′-bis­[3-(trimethylammonio)­propyl]­ferrocene dichloride (97%), basic blue 3, potassium ferrocyanide and **mCB**, using aqueous solutions of KCl (99%), KOH (98%) and NaOH (99%) salts as supporting electrolytes. High-purity nitrogen was used to obtain oxygen from the solutions. To prepare and characterize **mCB**, dimethyl sulfate (DMS) (99%), LiCl (99.9%), anhydrous acetonitrile, hexane (96%), diethyl ether (98%), 2-propanol (98%), dichloromethane (96%) and triethylamine (99%) compounds were also needed. All of the reagents were used as purchases.

### Cyclic Voltammetry Tests

Concentration and scan rate dependent experiments were performed using a multichannel SP-240 potentiostat/galvanostat from Biologic (France) with EC-Lab software. The experiments were carried out applying 95% of automatic IR drop compensation (Ru values ranging from 15 to 30 Ω) evaluated with the ZIR tool. The GC disk (*d* = 3 mm) working electrode was polished with 0.25 μm diamond powder (Büehler) and rinsed with distilled water. Commercial platinum wire and a Ag/AgCl aqueous electrode were used as auxiliary and reference electrodes, respectively.

### Flow Cell Tests

In-house made cells were used for charge–discharge the solutions. The system consists of two end plates supporting graphite and carbon cloth electrodes, separated by sheets of gasket (expanded, Teflon), sandwiching cation-exchange (Nafion 212) or anion-exchange (DSVN) membranes in the middle of the stacks. The liquids were pumped through Masterflex C-Flex tubings (Cole-Parmer, connected to the cell) with a Masterflex L/S, Cole-Parmer peristaltic pump. The flow cells were galvanostatically charged-discharged inside a glovebox using a LANHE battery testing system G340A at constant current density values of 20 and 5 mA cm^–2^.

### EPR Experiment

The ESR spectrum of reduced species of **mCB** in KCl solution was recorded in the X-band (9.4 GHz) at around 293 K (approximately 20 °C) using a MS5000X, Magnettech EPR/ESR spectrometer (Bruker) with an adjustment setup for microwave power frequency (10–20 mW), and magnetic field (200–650 mT) in quartz capillary (HIRSHMANN) which was then sealed with pliable plastic closure (Critoseal). The amplitude of the radical signal centered at *g* = 2.00 is off-scale together with the optimal experimental parameters. The resulting spectrum was processed by using ESRStudio software and Origin.

## Results and Discussion

### Alkaline Flow Battery Using **mCB** as a Two-Electron Storage *Negolyte*

To analyze phenoxazine aqueous FB electrolytes, a water-soluble **mCB** compound was prepared by *N*-methylation of **CB** with dimethyl sulfate (DMS), using acetonitrile (CH_3_CN) as reaction medium.^28^ The procedure is illustrated in Figure 2, and the product was filtered, washed, dried, and characterized by ^1^H NMR, ^13^C NMR, and HR-MS. These data and further details on the reaction procedure can be found in the Supporting Information (SI) section.

**Figure 2:**
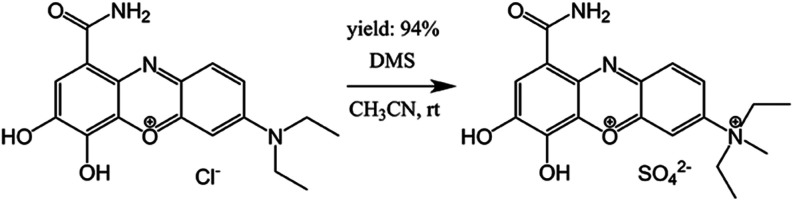
*N*-methylation illustration of **CB** (left) to obtain **mCB** (right).

The obtained proton NMR spectra of **CB** and **mCB** in D_2_O were analyzed in accordance with the aliphaticity and aromaticity. D-H exchange was seen on amide and phenolic protons on the molecule which were not elucidated due to invisibility. To compare aromatic and aliphatic proton peaks, the data were overlaid over the D_2_O peak around 4.7 ppm. After the methylation process and taking as a reference the starting material **CB**, it was seen that the aromatic proton peaks of **mCB** were respectively shifted to the low field which were more deshielded due to the slight change of phenoxazine ring’s aromaticity to become more negatively charged due to formed quaternary ammonium group (−NEt_2_Me^+^). The four aromatic protons are expected to be between 7.5 and 6.5 ppm. The three aliphatic protons of methyl and ethyl groups are also expected to be between 4.0 and 3.0 ppm. Furthermore, after the integration and peak comparison, ^1^H NMR spectra also show that the methylation occurred only on amino but not on hydroxyl groups.

The additional ^1^H- and ^13^C NMR spectra were also obtained in *d*_6_-DMSO. The ^1^H NMR spectrum of **mCB** in *d*_6_-DMSO showed very clear aromatic peaks between 7.8 and 6.9 ppm, and aliphatic protons of methyl and ethyl groups were seen around 3.9 and 3.4 ppm, respectively. Amide and phenolic protons were also visible as slightly broad peak around 5.4 ppm. The ^13^C NMR spectrum of **mCB** in *d*_6_-DMSO also showed that 16 different C atoms were laid between 165.2 and 41.8 ppm, from carboxamide to methyl­(ene) groups within downfield to upfield, respectively.

Next, ^1^H NMR and 2D-COSY NMR techniques were utilized (Figure 3) and program correlations were examined to determine the position of the methyl group. This analysis confirmed that the latter group is located at the quaternary nitrogen atom, ruling out monosubstituted methylation elsewhere. Initially, no correlation was observed between the methyl and ethyl groups attached to the tertiary ammonium group on the phenazine ring and the proton peaks in the aromatic region, which is the nature of the N centered system. This led to a detailed examination of the proton peaks in the aromatic region, as exemplified in Figure 3.

**Figure 3:**
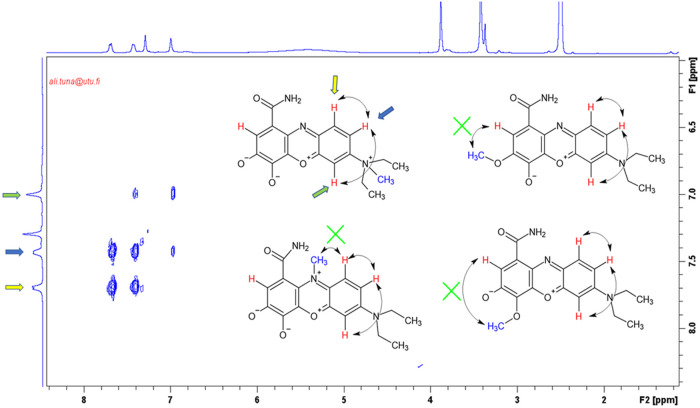
^1^H NMR based 2D-COSY NMR spectrum for **mCB** (Molecule 1) and four possible molecular structures with different positioned methyl substitutions (molecules 2, 3, and 4).

In the first molecule, the methyl group is on the nitrogen atom, and all relevant proton correlations match those in the experimental 2D-COSY NMR spectrum. In contrast, the second, third, and fourth molecules, where the methyl groups are bonded to different positions on the phenazine ring, do not show the necessary correlations around their systems. Therefore, it is concluded that the correct structure is represented by Molecule 1.

To analyze the viability of using the **mCB** (Figure 1) compound as an electroactive material for FB *negolytes*, we used **GAL** as a reference system and examined the reduction process of **mCB** and its starting material (**CB**) in KOH and NaOH solutions. The obtained voltammograms are shown in Figure 4. All tested phenoxazine derivatives exhibited a reversible two-electron reduction process as was expected.^1^ The differences in the peak current intensities (Figure 4A) of the voltammograms could result from different diffusion coefficients. The peak-to-peak potential distance of voltammograms (Δ*E*_p_ = *E*_pa_ – *E*_pc_, where *E*_pa_ and *E*_pc_ are the oxidation and reduction peaks, respectively) is dependent on the counterions K^+^ and Na^+^ (Figure 4B). To be consistent with Martínez et al.,^1^ the reversible **mCB** reduction process is followed by ion pairing with the cations and protonation reactions

1 $${\mathrm{mCB}}_{\mathrm{ox}}+2e^{-}⇋{\mathrm{mCB}}_{\mathrm{red}}^{2-},,E^{0},k_{s}$$

2 $${\mathrm{mCB}}_{red}^{2-}+nM^{+}⇋M_{n}{\mathrm{mCB}}_{red}^{n-2},,K_{ion}=k_{f1}/k_{b1}$$

3 $${\mathrm{mCB}}_{red}^{2-}+nH^{+}⇋H_{n}{\mathrm{mCB}}_{red}^{n-2},,K_{PT}=k_{f2}/k_{b2}$$

For testing the charge–discharge cycling stability of the target molecule in 1 mol L^–1^ KOH, we assembled a symmetric cell using 8 mL of salt solution containing 0.15 mol L^–1^ **mCB** compound as a *negolyte* and 12 mL of the same system in its reduced form (to have 0.15 mol L^–1^ of H_*n*_ **mCB**_red_^*n*–2^ species in the tank) as a *posolyte*. Both solutions were pumped through a homemade cell (composed of graphite and carbon cloth electrodes) with flat flow fields and separated with a Nafion 212 cation-exchange membrane. The experimentation was carried out inside a nitrogen-filled glovebox by applying current densities of ±20 mA cm^–2^ during 100 cell cycles (Figure 5A). When charging the cell, the electrons (two electrons per electroactive molecule, eqs 1–3) withdraw from the H_*n*_ **mCB**_red_^*n*–**2**^-KOH electrolyte and flow along the external circuit. As the latter solution was placed in excess, the **mCB**-KOH *negolyte* solution functioned as a capacity-limiting side.

**Figure 4:**
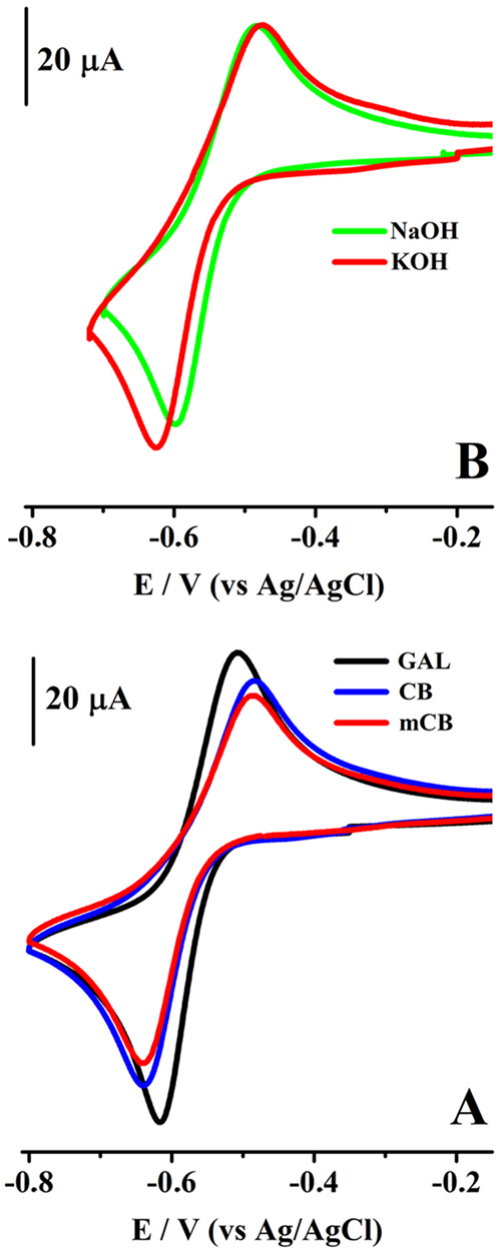
(A) Cyclic voltammograms for the reduction process of 0.0025 mol L^–1^ (**GAL**) gallocyanine, (**CB**) celestine blue, and (**mCB**) methyl celestine blue compounds studied in 1 mol L^–1^ KOH and (B: voltammograms for **mCB**) 1 mol L^–1^ NaOH (green line). Scan rate: 0.1 V s^–1^. WE: GC (*d* = 3 mm).

**Figure 5:**
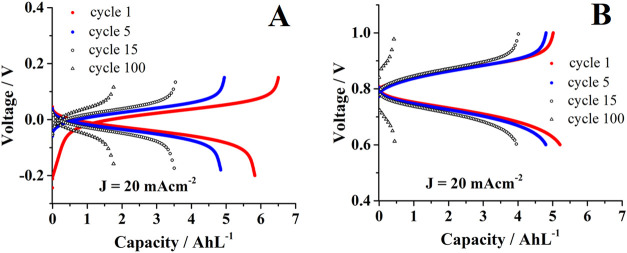
Representation of volumetric capacity as a function of cycle life for FBs: (A) symmetric cell assembled with 8 mL *negolyte* solution containing 0.15 mol L^–1^ **mCB** and 12 mL of the same solution in its reduced form (to have 0.15 mol L^–1^ of H_*n*_ **mCB**_red_^*n*–**2**^ species) in the *posolyte* tank; (B) 10 mL of the same *negolyte* composition paired to 20 mL of a *posolyte* solution containing 0.01 mol L^–1^ ferri- and 0.25 mol L^–1^ ferrocyanide species. One mol L^–1^ KOH was used as the supporting electrolyte and the cells were galvanostatic charging–discharging at a current density of 20 mA cm^–2^, using a cation-exchange membrane to separate the electrolytes.

From Figure 5A, a volumetric capacity of 6.54 AhL^–1^ is detected at the end of the first charging cycle, which is 81.34% of the theoretical capacity (8.04 AhL^–1^) for a *negolyte* material storing two electrons per electroactive molecule. So, this information reinforces the results presented above. The latter experimental value is higher than the one (1.72 AhL^–1^) reported for **GAL**-KOH *negolyte* ^1^ because **mCB** is more soluble (more than 0.15 mol L^–1^) than **GAL** (around 0.05 mol L^–1^) compound at alkaline conditions. However, upon discharging and continued cycling of the **mCB** based-cell, the capacity dropped to about 1.9 AhL^–1^ at the end of 100 cycles. The loss of capacity was due to partial precipitation of the electroactive material.

As it is reported,^29^ some phenoxazine molecules can interact between each other to form polymers by using −NH_2_ substituent groups as binding sites, but this process depends on the medium and pH, and during this, the amino group undergoes oxidation to create the binding site. In this connection, it is likely that −NH_2_ substituent groups promoted the aggregation of **mCB** reduced species and, consequently, their precipitation. In the symmetrical cell, it is not possible to assess species crossover during FB operation, or clearly determine whether the **mCB** molecule’s oxidation process becomes decisive.

To gather more information about the phenomenon, this *negolyte* was tested in a subsequent experiment against a *posolyte* composed of ferrocyanide and a small amount of potassium ferricyanide. The battery also shows a severe loss in capacity after a few cell cycles (Figure 5B); no crossover is observed in the cyclic voltammetry analysis of the charged-discharged electrolytes (Figure S15). The battery initially exhibits a significantly higher discharge capacity compared with its corresponding charging process (Figure 5B). In the subsequent cycles, the system behaves as expected, showing a lower discharge capacity. These results reinforce the idea that the oxidation process for the amino groups in **mCB** cannot be avoided during battery discharge, as it occurs under thermodynamic conditions similar to those of the process that controls cell operation. The voltammograms obtained (Figure S15) from this solution at the end of cycling show new signals resulting from the decomposition process. By the end of cycling, both solutions remained in their reduced state, suggesting that the *posolyte* tank must be rebalanced if further cycling is desired.

In summary, the target compound is more soluble than the **GAL** compound in KOH, but the cycling stability of the cell should be improved. It will be important to work in the molecular design of the **mCB** structure in order to prevent not only a possible crossover of species but also the formation of large precipitating aggregates between their reduced species.

### Reduction Mechanism of **mCB** in an Aqueous Neutral Condition

Taking advantage of the positively charged nature of structure **mCB**, we analyzed its electrochemistry in KCl solutions of neutral pH. As **GAL** and **CB** are partially soluble under this condition, basic blue 3 and 1,1′-bis­[3-(trimethylammonio)­propyl]­ferrocene chloride systems were also tested for comparisons. The obtained voltammograms are presented in Figure 6.

**Figure 6:**
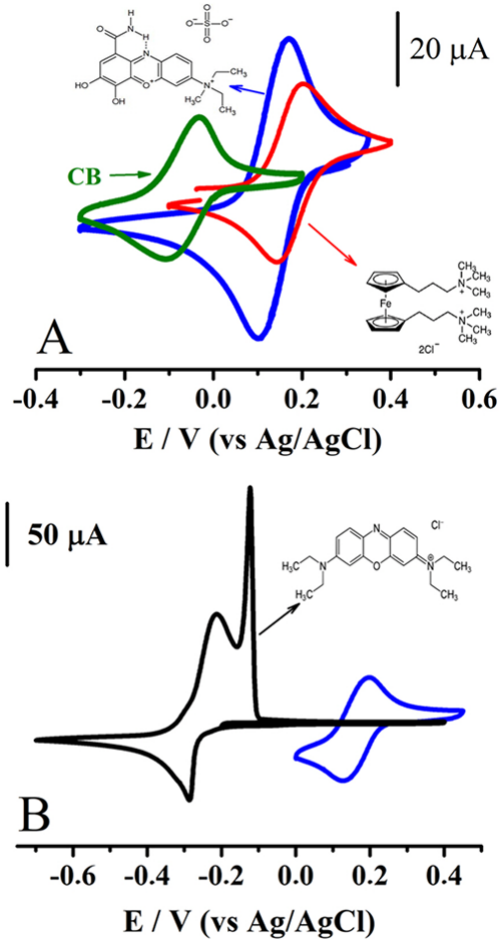
Cyclic voltammograms for the oxidation process of (red line) 1,1′-bis­[3-(trimethylammonio)­propyl]­ferrocene dichloride and the reduction process of (blue line) **mCB**, and (black line) basic blue 3 compounds in 1 mol L^–1^ KCl. Concentration of analyte: (A) 0.0025 mol L^–1^ and (B) 0.005 mol L^–1^. Scan rate of 0.1 V s^–1^. WE: GC (*d* = 3 mm).

As reported by Roushani and Karami,^30^ the starting material **CB** (in a concentration less than 0.001 mol L^–1^) undergoes a reversible two-electron reduction process in 0.1 mol L^–1^ phosphate buffer (pH 7) at −0.2 V (vs Ag/AgCl). We tested this compound in 1 mol L^–1^ KCl (green line) observing partial solubilization of the material and a redox potential of −0.1 V. Methylation of the latter compound (in this work) to prepare a **mCB** structure enhanced the solubility to around 0.1 mol L^–1^ in KCl and shifted the half-wave potential *E*_1/2_ = (*E*_pa_ + *E*_pc_)/2, characterizing the reversible reduction reactions taking place in KCl, to 0.15 V (vs Ag/AgCl, Figure 6A, blue line). This value is close to 0.19 V (vs Ag/AgCl, Figure 6A red line), the one exhibited by the typical *posolyte* solution used in neutral flow batteries: 1,1′-bis­[3-(trimethylammonio)­propyl]­ferrocene dichloride-KCl. The phenoxazine derivative is not highly soluble but is adequate to test its cyclic stability in a flow cell.

As can be seen from Figure 6, compound **mCB** has more substituent groups protecting its phenoxazine-based structure than the basic blue 3 derivative, and this protection helped the phenoxazine electrogenerated species not to strongly attach at the electrode surface (Figure 6B, black line versus blue line). CV of basic blue 3 shows typical behavior of surface deposition followed by a stripping peak during oxidation. This is interesting because to improve the reversibility in the case of phenoxazine basic blue 3 FB electrolyte,^23^ the authors incorporated super expensive electrocatalysts at the electrode surface, which is not the case here. Therefore, it is promising to analyze the stability of **mCB** electrogenerated species to propose its use as a new electroactive material for neutral *negolytes*.

As mentioned above, the reduction process of **mCB** involves the transfer of two-electrons (at a same thermodynamic condition) in KOH. The same goes for its starting material **CB** at neutral conditions.^30^ Although **mCB**-KCl can receive two electrons per electroactive molecule, assuming that it does so under the same thermodynamic conditions may not be correct. This is because the methylation of **CB** affects one of its redox centers to produce **mCB**. Also, the structure in neutral medium does not have the carbonyl redox center C=O formed at alkaline conditions. On the other hand, it should be considered that the same amount (0.0025 mol L^–1^) of **mCB** produces much more current than the system used as a reference (where it is known that one electron is transferred per molecule, Figure 6A, blue line versus red line), but perhaps not enough to consider the entry of two electrons. So, more information is required to decipher this electron transfer mechanism.

A proper strategy to analyze electron transfer reactions in phenoxazine FB electrolytes was previously reported,^1^ where it is recommended to perform cyclic voltammetry experiments as a function of scan rate (*v*), analyte concentration, and counterions present in the electrolyte (e.g., Na^+^, K^+^, and Li^+^). Some of these experiments were carried out, and the trends detected are reported below. As the half-wave potential and Δ*E*_p_ values (Figure 7A) of voltammograms exhibited marginal modifications by changing the positive charged counterions (Na^+^, K^+^, and Li^+^) in the electrolyte, ion pairing interactions with **mCB** reduced species are not significant in this case. This is reasonable because, regardless of whether the structure **mCB** receives one or two electrons, it does not acquire negative charges that favor its interaction with positively charged counterions as in alkaline conditions.

**Figure 7:**
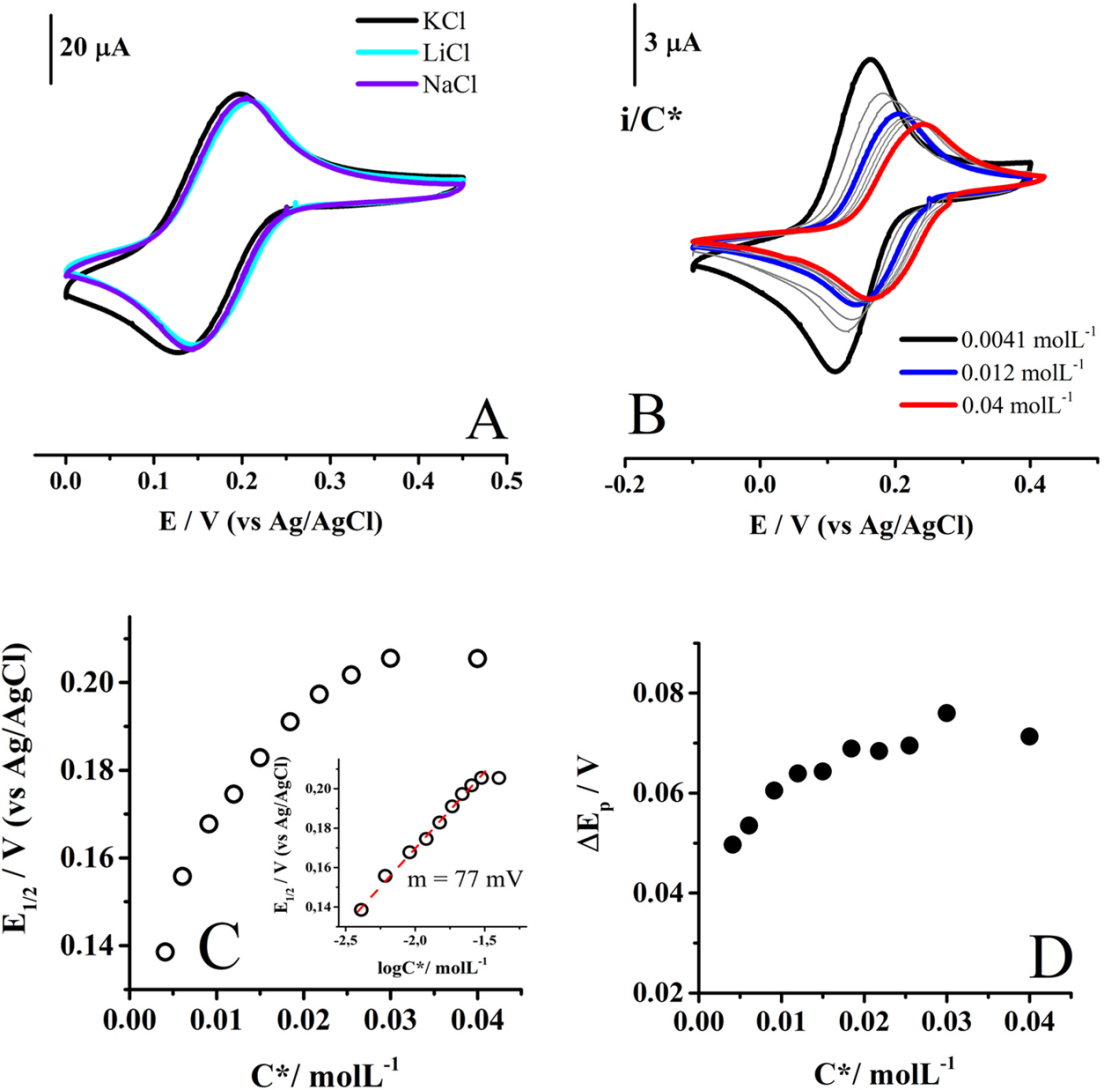
(A) Cyclic voltammograms for the first reduction process of 0.025 mol L^–1^ **mCB** compound in aqueous solutions having 1 mol L^–1^ of different neutral salts. (B) **mCB** concentration dependent experiments in 1 mol L^–1^ KCl, where the signals are normalized versus C*. Variations of (C) *E*_1/2_ and (D) Δ*E*_p_ as a function of C*, where m is the slope of the fitting. Scan rate of 0.1 V s^–1^. WE: GC (*d* = 3 mm).

An interesting transition appeared when normalizing the current values of the voltammograms obtained as a function of analyte concentration C* (Figure 7B). Upon increasing amounts of C*, the signal apparently exhibited less current than expected, but this trend changed when reaching 0.012 mol L^–1^ of **mCB** in solution. From this value and by further increasing C*, the system seems to behave as predicted by Randles-Sevcik equation for diffusion-controlled mechanisms,^31^ where the normalized current values are independent of C*.

Increasing C* in solution also shifted *E*_1/2_ (Figure 7C) toward positive values and this thermodynamic behavior could indicate the occurrence of a second order mechanism.^31,32^ Since the system does not lose reversibility during increases in C*, the second order process could also be fast and reversible. This phenomenon is typically detected in electron transfer controlled hydrogen bonding processes (ETCHB), where the concentration of a proton donor is increased while radical proton donor–acceptors are electrogenerated.^33^ In our system, protons can come from water, but their concentration does not change, so the thermodynamic effects detected when increasing C* are not related to ETCHB processes.

The electrochemical behavior of mCB compound (Figure 7B) is similar to that reported for viologen FB electrolytes, where their electrochemical behavior was related to the occurrence of a fast and reversible dimerization process between reduced species.^34^ In fact, this is the only second order bimolecular-mechanistic option that can produce large shifts of *E*_1/2_ as a function of C*, without observing important changes in the shape of voltammograms (although this would only apply to concentrations greater than 0.012 mol L^–1^ of **mCB**):^35^ *E*_1/2_ varies linearly with log C* at a rate of 30/*n* mV per unit log C*. To examine this possibility, in this work, we plotted *E*_1/2_ data versus log C* (Figure 7C) and found a slope of 77/*n* mV, which is much larger than 30 and 15 mV for one and two electron transfers, respectively. From the resulting graph, we also realized that the system reaches a limit value where *E*_1/2_ no longer moves, which is not in line with the occurrence of a fast and reversible dimerization process. Therefore, another mechanistic proposal should be explored to explain the electrochemistry of **mCB** in KCl.

Considering another analysis strategy, the dependence of Δ*E*_p_ as a function of C* was examined (Figure 7D), detecting a change in the magnitude of Δ*E*_p_ from 49 to 75 mV upon increasing C* from 0.0041 to 0.04 mol L^–1^. This behavior is not exhibiting a kinetically controlled process because the voltammograms are not significantly losing current when Δ*E*_p_ tends to be larger. Then, it would appear that the signal is transitioning from a bielectronic wave (Δ*E*_p_ < 60 mV) to a monoelectronic one (Δ*E*_p_ > 60 mV),^31,32^ but this appears to happen immediately after the first increases in C* (from 0.0041 to 0.012 mol L^–1^) for which there seems to be no reasonable explanation.

To get more insights into the reduction mechanism of **mCB**-KCl electrolyte, the system was scanned at potential values as negative as possible. The voltammogram obtained (Figure 8) revealed a second irreversible reduction process occurring at −1.42 V (vs Ag/AgCl); its wave exhibited a maximum peak current intensity similar to that exhibited by the signal detected at 0.15 V (vs Ag/AgCl). This, in fact, reveals that both processes involve the same number of transferred electrons. Thinking that each molecule of **mCB** can receive 4 electrons per molecule is inconsistent, and therefore, it is proposed that the system undergoes a first reversible reduction process (in KCl) receiving one electron per electroactive molecule to form **mCB**_red_^**+**•^ radicals. Note that electrogenerated species could evolve into a protonation process, but it is also likely that their positive charge prevents this reaction. For practical issues, the formation of the radical is simplified by eq 4. However, it is still necessary to explain the transitions detected with varying amounts of C*.

4 $${\mathrm{mCB}}_{\mathrm{ox}}^{2+}+e^{-}⇋{\mathrm{mCB}}_{\mathrm{red}}^{+•},,E^{0},k_{s}$$

For analyzing the transition detected between 0.0041 and 0.012 mol L^–1^ (Figure 7), we examined the scan rate dependence of the system at 0.005 mol L^–1^ **mCB**, and the voltammograms obtained are presented in Figure 9.

**Figure 8:**
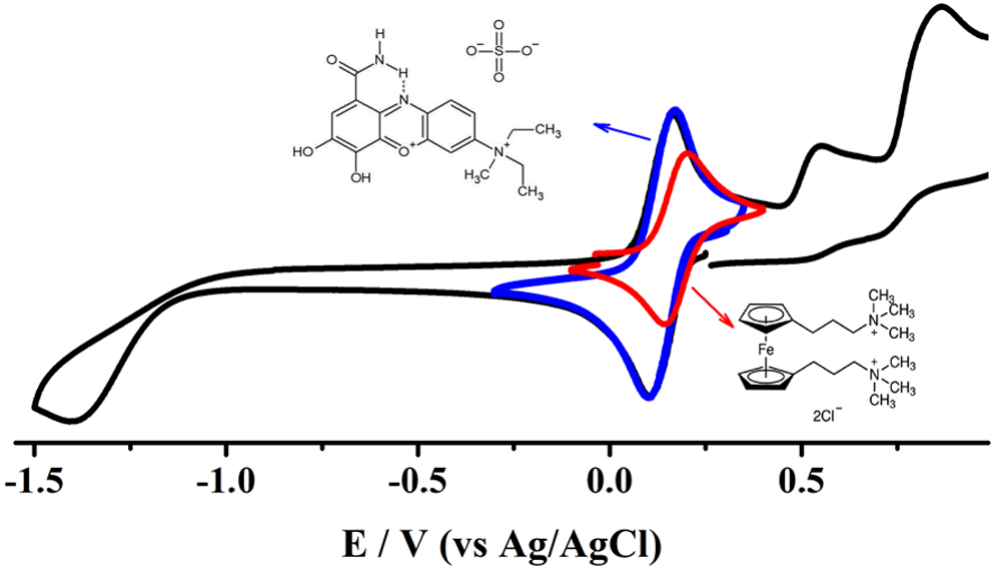
Cyclic voltammograms for the first (blue line) and second (black line) reduction processes of **mCB** and for the oxidation process of (red line) 1,1′-bis­[3-(trimethylammonio)­propyl]­ferrocene dichloride in 1 mol L^–1^ KCl. Concentration of analyte 0.0025 mol L^–1^, scan rate 0.1 V s^–1^. WE: GC (*d* = 3 mm).

**Figure 9:**
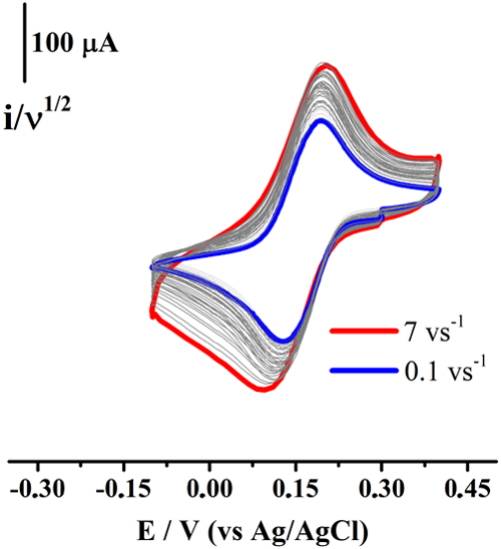
Cyclic voltammograms for the reduction process of 0.005 mol L^–1^ **mCB** compound studied in 0.5 mol L^–1^ KCl, as a function of scan rate *v*. WE: GC (*d* = 3 mm).

The normalized voltammograms (versus *v* ^–1/2^) exhibited higher current values and the magnitude of Δ*E*_p_ was modified very slightly when increasing the scan rate. The electrochemistry detected is similar to the one reported for the starting material **CB** and is consistent with the occurrence of a mixed and reversible outer- and inner-sphere electron transfer mechanism,^1,30,36^ where, in the first stage (before occurring the charge transfer process), some **mCB** molecules are covalently attached to the electrode surface (forming layers) without passivating it. For the starting material **CB**, the process involves the transfer of two electrons per electroactive molecule, and Δ*E*_p_ = 20 mV at low concentrations of C* less than 0.001 mol L^–1^.^30^ This is because under this condition, the response is predominantly that of a surface bound species, but the response should become more diffusional in nature as C* increases.^36^ In that sense, one way to evidence these types of mixed mechanisms is by measuring the increase in the magnitude of Δ*E*_p_ with concentration C*, which now explains the transition detected for the **mCB** compound in KCl (Figure 7D). Then, eqs 4 and 5 represent the reactions taking place at the electrode surface at high and low concentrations of C*, respectively.

5 $${\mathrm{mCB}}_{\mathrm{ox}\left(\mathrm{ads}\right)}^{2+}+e^{-}⇋{\mathrm{mCB}}_{\mathrm{red}\left(\mathrm{ads}\right)}^{+•},,E2^{0},k2_{s}$$

In this type of mixed mechanisms, the redox potential is depending on the electrode material.^1^ So, at low concentrations of **mCB**, the electronic properties of the glassy carbon material also control the thermodynamics of the system, but things are different at high C* concentrations: the redox potential is depending on the electronic properties of the already modified electrode with **mCB** molecules, which makes the process behave diffusive. This is the reason why the voltammetric wave for **mCB** moves toward positive values as C* increases and stops when it reaches the new thermodynamic condition, where the energetic contribution of the interactions between the species transformed and the electrode became negligible. These results highlight the importance of studying FB electrolytes as a function of scan rate and analyte concentration before testing flow batteries. With this, it is possible to properly deduce the electron transfer mechanism of the system, the electrons transferred and the diffusion coefficient. It also allows avoiding comparisons between results obtained from inner-sphere electron transfer processes with those obtained from outer-sphere electron transfer mechanisms. Therefore, the voltammetric behavior detected for the **mCB** compound in KCl as a function of scan rate and C* is related to a mixed mechanism involving outer- and inner-sphere one-electron reduction reactions to produce stable radical cations (eqs 4 and 5).

### Neutral Redox Flow Battery Using **mCB** as a *Posolyte*

To test the cycling stability of the cation radicals formed upon reducing **mCB** compound (eq 4) in 0.4 mol L^–1^ KCl, we assembled a symmetric cell using 15 mL of salt solution containing 0.04 mol L^–1^ **mCB** compound as a *negolyte* and 20 mL of the same system in its reduced form (to have 0.04 mol L^–1^ of **mCB**_red_^**+**•^ species in the tank) as a *posolyte*. Although the solubility of the compound is close to 0.1 mol L^–1^ in 1 mol L^–1^ KCl, we used lower concentrations of salt and **mCB** in order to avoid the precipitation of electrogenerated species. The solutions were separated by a selenium DSVN anion-exchange membrane, and the cell was tested inside a glovebox by applying current densities of ±5 mA cm^–2^ (Figure 10). When charging the cell, the electrons (one electron per electroactive molecule) are withdrawn from electrolyte **mCB**_red_^**+**•^-KCl, while *negolyte* solution **mCB**-KCl functioned as a capacity-limiting side.

**Figure 10:**
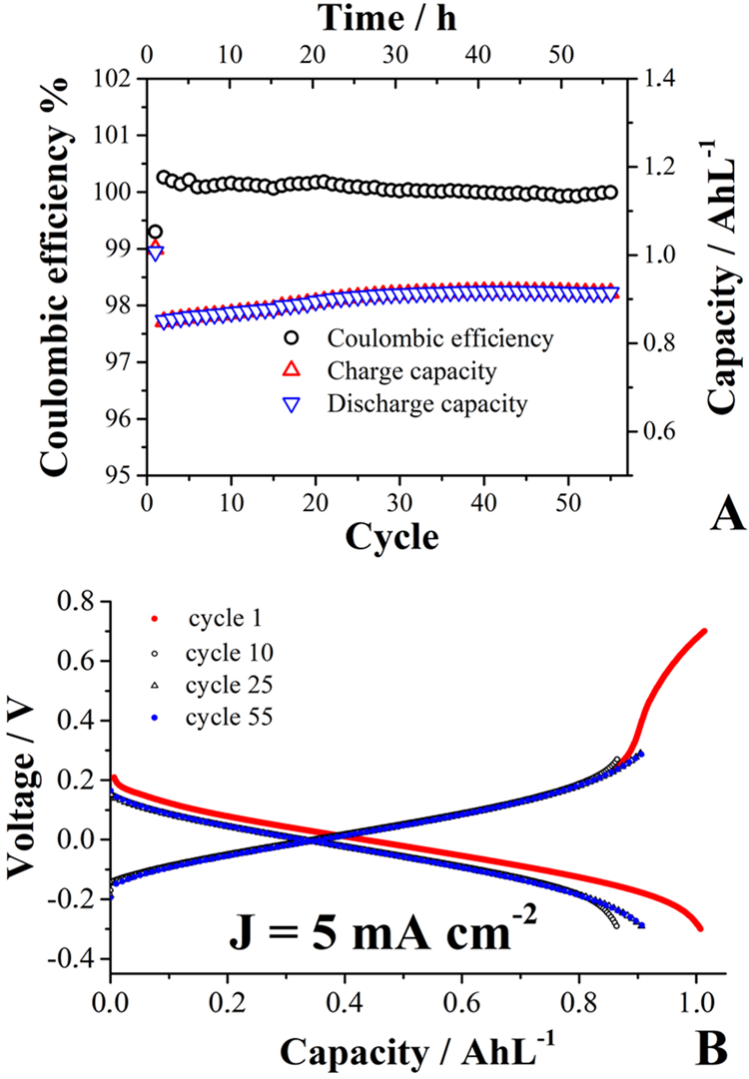
Galvanostatic cycling parameters (A) for a symmetric cell assembled with 15 mL *negolyte* solution containing 0.04 mol L^–1^ **mCB** in 0.4 mol L^–1^ KCl and 20 mL of the same solution in its reduced form (to have 0.04 mol L^–1^ of **mCB**_red_^**+**•^ species in the *posolyte* tank), operated at a constant current density of 5 mA cm^–2^, using an anion-exchange membrane. (B) Representation of the capacity retention as a function of cycling life. The cutoff voltage for the discharging process was set at −0.3 V, while the corresponding to the discharging process was increased after passing 20 cycles from 0.27 to 0.3 V.

The symmetric cell displayed (Figure 10A) a Coulombic efficiency of >99% and a significant long cycling lifetime. From Figure 10B, a volumetric capacity of 1.02 AhL^–1^ is detected during the first charge–discharge cell cycle, which is 95% of the theoretical capacity (1.07 AhL^–1^). However, some kinetic limitations (probably because of the adsorption abilities of the compound) were observed when the total storage capacity of the material. Therefore, the cell was continue cycled considering a maximum capacity (0.92 AhL^–1^) of 86% with respect to the theoretical capacity and the system maintained full storage capacity after 55 charge–discharge cell cycles. This result is in line with those presented in the previous section and supports the proposal that **mCB**_red_^**+**•^ radicals are formed during the one-electron reduction process of **mCB** in KCl. To demonstrate the formation of radical species, we stopped the cycling experiment after charging the target electrolyte (the capacity-limiting side) to 95% of its theoretical capacity and analyzed the solution by EPR, detecting a strong radical signal with hyperfine coupling interactions (Figure 11).

**Figure 11:**
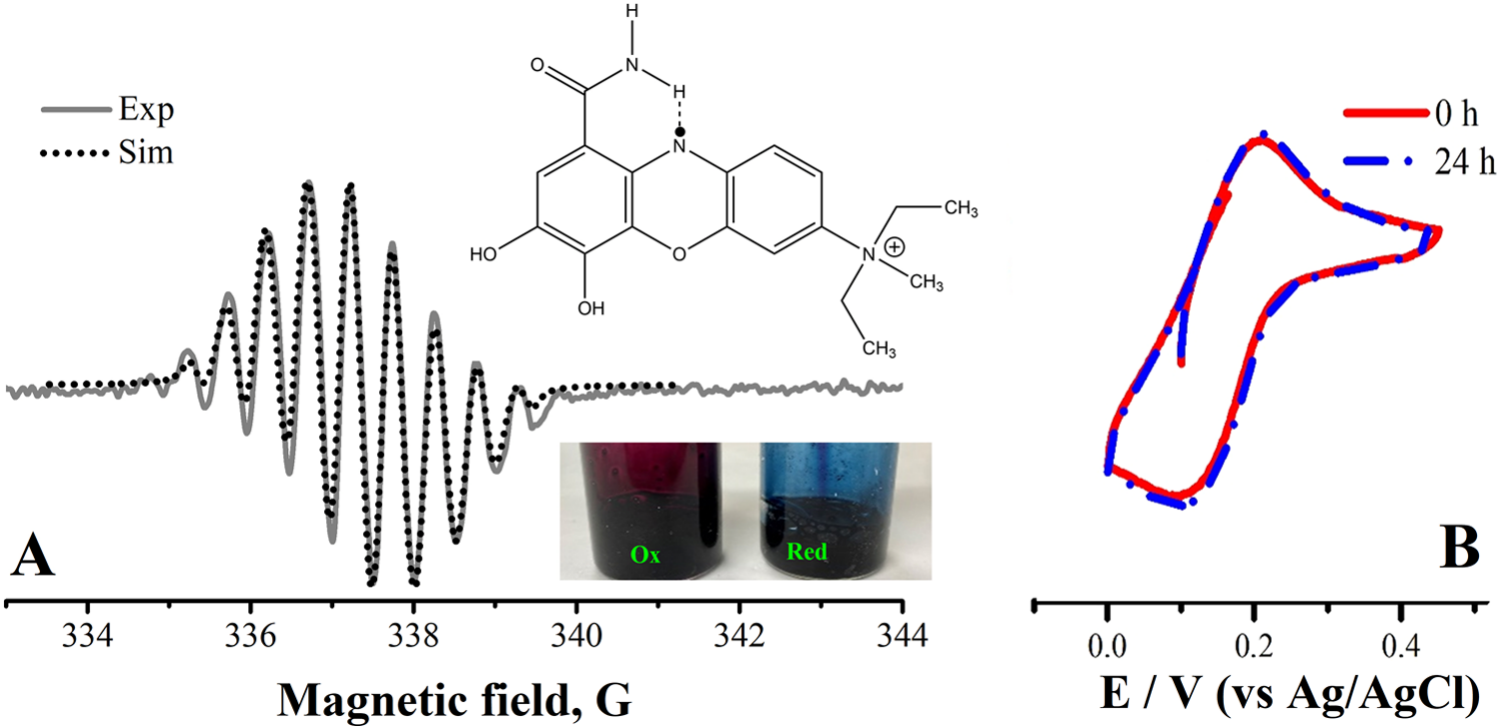
(A) EPR and (B) cyclic voltammetry experiments for the reduced form of compound **mCB** to form **mCB**_red_^**+**•^, after 55 charge–discharge cycles in the symmetric FB cell. The experiments were carried out without bubbling nitrogen and after taking the solution out of the glovebox. The hyperfine coupling constants used for the EPR simulation are the following: AN1 = 5.8; AN2 = 5.4; AH1 = 4.9; AH2 = 4.6; AH3 = 4.4; AH4 = 3.8; AH5 = 1.

As commented in the Introduction section, electrogenerated phenoxazine species have been reported to be unstable and short-lived in neutral aqueous FB electrolytes, due to some decomposition mechanisms.^10^ In this case, the EPR spectrum of **mCB**_red_^**+**•^ species was recorded (Figure 11A) after 8 h of taking the tank out of the glovebox and the solution was also monitored as a function of time by cyclic voltammetry (Figure 11B), without bubbling nitrogen in the cell. As marginal changes in the CV wave of the solution were detected after 24 h, the global results from cyclic voltammetry, battery test, and EPR suggest that radical species **mCB**_red_^**+**•^ formed when reducing **mCB** in KCl shows a certain level of stability against some traces of oxygen. Future time-dependent ERP studies could confirm the air-stability of this radical. From simulated EPR, hyperfine coupling constants (of similar magnitude) for various N and H atoms were obtained, which means that the radical can be delocalized within a large part of the skeleton, **mCB**_red_^**+**•^. This type of resonance stabilization was also detected in other aromatic compounds.^13,37,38^ Motivated by these results, we decided to test a 0.73 V FB by coupling this *posolyte* to a KCl solution containing a methyl viologen derivative as a *negolyte*. The results obtained are presented in Figure 12.

**Figure 12:**
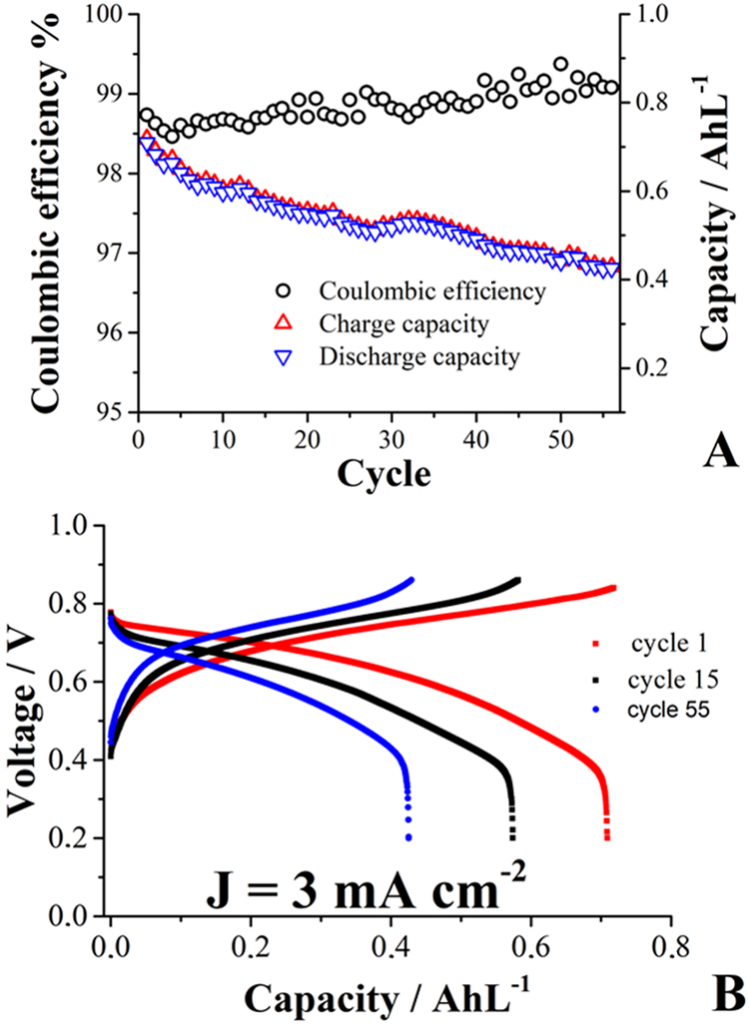
Galvanostatic cycling parameters (A) for a 0.73 V FB cell operated with 10 mL of an aqueous *posolyte* solution containing 0.03 mol L^–1^ **mCB**_red_^**+**•^ in 1 mol L^–1^ KCl. Excess solution of 0.06 mol L^–1^ 1,1′-bis­[3-(trimethylammonio)­propyl]-4,4′-bipyridinium tetrabromide (viologen) was used as a *negolyte*. (B) Capacity retention as a function of cycling life, including a representation of the detected charge–discharge voltage profiles detected. Initial capacity utilization of the *posolyte* was 0.72 AhL^–1^, which is 90.5% of the theoretical capacity, 0.8 AhL^–1^.

As the battery lost 41% of its initial capacity after a few charge–discharge cell cycles, the proposal of using several substituent groups for protecting phenoxazine-based structures remains insufficient. Unlike the less protected phenoxazines studied at pH 7 in previous work,^10,27^ , no new signals emerged, nor were there any significant changes in the shape of the voltammograms from the cycled solutions in our study (Figure S16), indicating that serious degradation of the material did not occur at the methyl celestine blue structure. Therefore, we propose that significant improvement will be done through molecular designing, for instance, by changing the nature of the substituents. Since no crossover of **mCB** species was detected (based on the analysis of the voltammograms obtained from the separated solutions) at the end of the experiment, the loss in capacity is probably related to an oxidation process of the amino substituent group of **mCB**, since this process occurs at a potential value very close to that of the main process (Figure 8). As released protons from the oxidation of amino groups can move freely across the membrane, they may be responsible for lowering the pH in the *negolyte* tank from 7 (at cycle 0) to 4 (after 55 cycles). Note that CE is less than 99% in the first ten cycles, then it is likely that the *negolyte* solution is also acidified due to the electrolysis of water in this tank, although this process should not be very quantitative and due to the low redox potential of viologens.^39^

Another observation that caught attention is the detection of a significant amount of electroactive material in the electrode materials, which is also consistent with cyclic voltammetry experiments that exhibited a mixed mechanism composed of outer- and inner-sphere electron transfer reactions. Under this condition, conventional methods typically considered in FB applications (e.g., Nicholson approach, etc.) for obtaining kinetic and thermodynamic information cannot be applied in the analysis of our CV responses. Therefore, apparent values of diffusion coefficient (*D*_o_) and electron transfer rate constant (*k*_s_) were estimated by chronoamperometry (Figure S17) and fitting voltammetric simulations (Figure S18) to experimental data, respectively. The results obtained are comparable with other values reported for fast and reversible electron transfer processes:^10,27^ *k*_s_ = 0.05 cm s^–1^, *D*_o_ = 5.76 × 10^–6^ cm^2^ s^–1^. For future work, it will be useful to work on the molecular design of **mCB** to improve its stability and solubility to obtain a higher tank volumetric capacity. An interesting approach utilize additives and molecular spectators, as was demonstrated in a previous work when improving the volummetric capacity of **GAL** FB electrolyte.^40^ The results obtained here could also be relevant for the design of phenoxazine compounds with biological activity, as water-stable radicals with high resistance to oxygen are the most promising systems for this purpose.^22,25^

## Conclusions

The study of water-soluble phenoxazine electroactive materials is crucial for improving the development of FB electrolytes, especially on the positive side. Herein, the electron transfer process of commercially available phenoxazine derivatives was investigated in KCl solutions.

A water-soluble phenoxazine structure (**mCB**) was obtained by methylating compound **CB**. The synthesized structure exhibited a relatively high redox potential (0.2 V vs Ag/AgCl), reversible redox chemistry, and full capacity retention when tested as an electroactive material in a symmetrical FB cell, during 55 charge–discharge cell cycles.

The reduction process in compound (**mCB**) involves the transfer of one electron per electroactive molecule to form the radical species **mCB**_red_^**+**•^ as evidenced by cyclic voltammetry and electron paramagnetic resonance spectroscopy. Its unusual voltammetric behavior exhibited a mixed mechanism composed of outer- and inner-sphere electron transfer reactions. For properly elucidating the reaction mechanism and kinetics of FB electrolytes, a more exhaustive characterization by cyclic voltammetry is suggested. The loss of battery performance capacity detected for this electrolyte paired with a viologen-based *negolyte* was related to an oxidation process of amino substituents contained in the **mCB** structure, which delivers protons to the solution lowering the pH from 7 to 4.

## Supplementary Material



## Notes

Molecular spectral analyses (NMR, high resolution mass, IR, UV–vis, EPR), electrochemical characterizations (cyclic voltammograms, plots, simulations, etc.), and battery tests (charge/discharge cycles) (PDF)

## References

[1] Martínez-González E., Amador-Bedolla C., Ugalde-Saldivar V. M. (2022). Reversible Redox Chemistry in a Phenoxazine-Based
Organic Compound: A Two-Electron Storage Negolyte for Alkaline Flow
Batteries. ACS Appl. Energy Mater..

[2] Kwabi D. G., Ji Y., Aziz M. J. (2020). Electrolyte
lifetime in aqueous organic redox flow
batteries: a critical review. Chem. Rev..

[3] Singh V., Kim S., Kang J., Byon H. R. (2019). Aqueous organic redox flow batteries. Nano Res..

[4] Nolte O., Volodin I. A., Stolze C., Hager M. D., Schubert U. S. (2021). Trust is
good, control is better: a review on monitoring and characterization
techniques for flow battery electrolytes. Mater.
Horiz..

[5] Kwabi D. G., Lin K., Ji Y., Kerr E. F., Goulet M.-A., De Porcellinis D., Tabor D. P., Pollack D. A., Aspuru-Guzik A., Gordon R. G. (2018). Alkaline quinone flow battery with long lifetime
at pH 12. Joule.

[6] Lv Y., Liu Y., Feng T., Zhang J., Lu S., Wang H., Xiang Y. (2019). Structure
reorganization-controlled electron transfer of bipyridine
derivatives as organic redox couples. J. Mater.
Chem. A.

[7] DeBruler C., Hu B., Moss J., Liu X., Luo J., Sun Y., Liu T. L. (2017). Designer two-electron
storage viologen anolyte materials
for neutral aqueous organic redox flow batteries. Chem.

[8] Lin K., Gómez-Bombarelli R., Beh E. S., Tong L., Chen Q., Valle A., Aspuru-Guzik A., Aziz M. J., Gordon R. G. (2016). A redox-flow battery
with an alloxazine-based
organic electrolyte. Nat. Energy.

[9] Zhang C., Niu Z., Peng S., Ding Y., Zhang L., Guo X., Zhao Y., Yu G. (2019). Phenothiazine-based
organic catholyte
for high-capacity and long-life aqueous redox flow batteries. Adv. Mater..

[10] Fang X., Zeng L., Li Z., Robertson L. A., Shkrob I. A., Zhang L., Wei X. (2023). A cooperative
degradation
pathway for organic phenoxazine catholytes in aqueous redox flow batteries. Next Energy.

[11] Beh E. S., De Porcellinis D., Gracia R. L., Xia K. T., Gordon R. G., Aziz M. J. (2017). A neutral pH aqueous organic-organometallic
redox flow
battery with extremely high capacity retention. ACS Energy Lett..

[12] Chen Y., Zhou M., Xia Y., Wang X., Liu Y., Yao Y., Zhang H., Li Y., Lu S., Qin W. (2019). A stable and high-capacity redox targeting-based electrolyte
for
aqueous flow batteries. Joule.

[13] Zhang Y., Li F., Li T., Zhang M., Yuan Z., Hou G., Fu J., Zhang C., Li X. (2023). Insights into an air-stable methylene
blue catholyte towards kw-scale practical aqueous organic flow batteries. Energy Environ. Sci..

[14] Rubio-Presa R., Lubián L., Borlaf M., Ventosa E., Sanz R. (2023). Addressing
Practical Use of Viologen-Derivatives in Redox Flow Batteries through
Molecular Engineering. ACS Mater. Lett..

[15] Kosswattaarachchi A.
M., Cook T. R. (2018). Repurposing
the industrial dye methylene blue as an
active component for redox flow batteries. ChemElectroChem.

[16] Hasan F., Mahanta V., Abdelazeez A. A. A. (2023). Quinones
for Aqueous Organic Redox
Flow Battery: A Prospective on Redox Potential, Solubility, and Stability. Adv. Mater. Interfaces.

[17] Yang B., Hoober-Burkhardt L., Krishnamoorthy S., Murali A., Prakash G. S., Narayanan S. (2016). High-performance
aqueous organic flow battery with
quinone-based redox couples at both electrodes. J. Electrochem. Soc..

[18] Pang S., Li L., Ji Y., Wang P. (2024). A Multielectron and High-Potential
Spirobifluorene-Based Posolyte for Aqueous Redox Flow Batteries. Angew. Chem., Int. Ed..

[19] Ge G., Mu C., Wang Y., Zhang C., Li X. (2025). Four-Electron-Transferred
Pyrene-4, 5, 9, 10-tetraone Derivatives Enabled High-Energy-Density
Aqueous Organic Flow Batteries. J. Am. Chem.
Soc..

[20] Fan H., Wu W., Ravivarma M., Li H., Hu B., Lei J., Feng Y., Sun X., Song J., Liu T. L. (2022). Mitigating
ring-opening to develop stable TEMPO catholytes for pH-neutral all-organic
redox flow batteries. Adv. Funct. Mater..

[21] Liu Y., Goulet M.-A., Tong L., Liu Y., Ji Y., Wu L., Gordon R. G., Aziz M. J., Yang Z., Xu T. (2019). A long-lifetime
all-organic aqueous flow battery utilizing TMAP-TEMPO radical. Chem.

[22] Li L., Su Y., Ji Y., Wang P. (2023). A Long-Lived Water-Soluble Phenazine
Radical Cation. J. Am. Chem. Soc..

[23] Li H., Fan H., Ravivarma M., Hu B., Feng Y., Song J. (2020). A stable organic
dye catholyte for long-life aqueous flow batteries. Chem. Commun..

[24] Otteny F., Perner V., Wassy D., Kolek M., Bieker P., Winter M., Esser B. (2020). Poly (vinylphenoxazine) as fast-charging
cathode material for organic batteries. ACS
Sustainable Chem. Eng..

[25] Onoabedje E. A., Egu S. A., Ezeokonkwo M. A., Okoro U. C. (2019). Highlights of molecular
structures and applications of phenothiazine & phenoxazine polycycles. J. Mol. Struct..

[26] Lee K., Serdiuk I. E., Kwon G., Min D. J., Kang K., Park S. Y., Kwon J. E. (2020). Phenoxazine
as a high-voltage p-type
redox center for organic battery cathode materials: small structural
reorganization for faster charging and narrow operating voltage. Energy Environ. Sci..

[27] Qin M., Wu G., Zheng K., Yu X., Xu J., Cao J. (2024). A highly water-soluble
phenoxazine quaternary ammonium compound catholyte for pH-neutral
aqueous organic redox flow batteries. J. Energy
Storage.

[28] Prashad M., Har D., Hu B., Kim H.-Y., Repic O., Blacklock T. J. (2003). An efficient
and practical N-methylation of amino acid derivatives. Org. Lett..

[29] Schlereth D. D., Karyakin A. A. (1995). Electropolymerization of phenothiazine, phenoxazine
and phenazine derivatives: characterization of the polymers by UV–visible
difference spectroelectrochemistry and Fourier transform IR spectroscopy. J. Electroanal. Chem..

[30] Roushani M., Karami E. (2014). Electrochemical Detection of Persulfate
at the Modified
Glassy Carbon Electrode with Nanocomposite Containing Nano-Ruthenium
Oxide/Thionine and Nano-Ruthenium Oxide/Celestine Blue. Electroanalysis.

[31] Bard, A. J. ; Faulkner, L. R. Electrochemical Methods: Fundamentals and Applications; Wiley: New York, 2001.

[32] Savéant, J.-M. Elements of Molecular and Biomolecular Electrochemistry: An Electrochemical Approach to Electron Transfer Chemistry; John Wiley & Sons, 2006.

[33] Martínez-González E., Frontana C. (2014). Inner reorganization
limiting electron transfer controlled
hydrogen bonding: intra-vs. intermolecular effects. Phys. Chem. Chem. Phys..

[34] Martinez-Gonzalez E., Flores-Leonar M. M., Amador-Bedolla C., Ugalde-Saldivar V. M. (2021). Concentration
effects on the first reduction process of methyl viologens and diquat
redox flow battery electrolytes. ACS Appl. Energy
Mater..

[35] Savéant J., Vianello E. (1967). Potential-sweep voltammetry: theoretical analysis of
monomerization and dimerization mechanisms. Electrochim. Acta.

[36] Batchelor-McAuley C., Gonçalves L. M., Xiong L., Barros A. A., Compton R. G. (2010). Controlling
voltammetric responses by electrode modification; using adsorbed acetone
to switch graphite surfaces between adsorptive and diffusive modes. Chem. Commun..

[37] S
V S. S., John P. C. S., Paton R. S. (2021). A quantitative metric for organic
radical stability and persistence using thermodynamic and kinetic
features. Chem. Sci..

[38] Tang B., Zhao J., Xu J.-F., Zhang X. (2020). Tuning the stability
of organic radicals: from covalent approaches to non-covalent approaches. Chem. Sci..

[39] Hu B., DeBruler C., Rhodes Z., Liu T. L. (2017). Long-cycling aqueous
organic redox flow battery (AORFB) toward sustainable and safe energy
storage. J. Am. Chem. Soc..

[40] Martínez-González E., Peljo P. (2024). Improving the Volumetric Capacity of Gallocyanine Flow Battery by
Adding a Molecular Spectator. ACS Appl. Energy
Mater..

