# Supplementary material for: Phenoxazine Radical as a Positive Material for Neutral pH Aqueous Flow Batteries
Source: ACS Appl Energy Mater. 2025 May 5;8(10):6463–73. doi: 10.1021/acsaem.5c00225 (PMC12117522; doi:10.1021/acsaem.5c00225)
Supplement: Supplementary file 1 [file ae5c00225_si_001.pdf]

Supporting Information

Phenoxazine Radical as Positive Material for Neutral pH Aqueous Flow Battery

Eduardo Martínez-González,*[a] Ali Tuna[a,b], and Pekka Peljo*[a]

[a]University of Turku, Department of Mechanical and Materials Engineering, Vesilinnantie 5, Turku, FI-20014, Finland
[b]University of Turku, Department of Chemistry, Henrikinkatu 2, Turku, FI-20014, Finland

E-mail: edumg109@gmail.com; pekka.peljo@utu.fi

Synthesis of methyl celestine blue (MCB)

To a suspension of celestine blue (9.823 g, 27 mmol, 1 eq) in acetonitrile (125 mL), dimethylsulfate (DMS, 25 mL, 263.62 mmol, 9.75 eq) was added dropwise. The reaction mixture was stirred for 48 hours at room temperature. Afterwards, the reaction mixture was poured onto diethyl ether (250 mL) and the solid was collected by paper-filtration at room temperature. The crude was suspended in slightly warmed hexane (150 mL) and then hot-filtered again at 60-65°C. The dark solid was washed with acetonitrile, isopropanol and dichloromethane, around 50 to 55 mL each respectively to remove unreacted starting material of celestine blue without needing a column chromatography. The solid was dried under reduced pressure in a hot water bath for overnight at 75–80˚C to give methyl celestine blue as a dark powder (11.153 g, 94%). The obtained solid was characterized by relevant analytical techniques. The melting points for the starting material CB and the product mCB are 225-230°C and 210-215°C, respectively.

Note: The unreacted dimethyl sulfate was further quenched with the mixture of excess triethylamine and isopropanol to terminate the alkylating power.

^{1}H NMR (500 MHz, 298 K, D_2O, ppm): δ= 7.33–7.00 (m, 3H), 6.54 (s, 1H), 3.86 (s, 6H, -CH_3), 3.50 (s br, 4H, CH_2); ^{1}H NMR (500 MHz, 298 K, d_6-DMSO, ppm): δ= 7.69 (d, 1H, J= 8.5 Hz), 7.43 (d, 1H, J= 7 Hz), 7.29 (s, 1H), 5.42 (s br, 4H, NH_2 & OH), 3.88 (s, 4H, CH_2), 3.42 (s, 6H, CH_3), 3.37 (s, 3H, CH_3); ^{13}C NMR (125 MHz, 298 K, d_6-DMSO, ppm): δ= 165.17, 162.10, 157.84, 147.73, 135.58, 134.96, 134.05, 133.47, 132.43, 127.43, 120.78, 118.94, 97.01, 52.81, 52.63, 41.89; HRMS (ESI): calcd for [M]: 343.1532 Da, found: 343.1510 Da, *m/z* calcd for [M+K^{+}]: 382.1169, found: 382.1141

Structural analyses

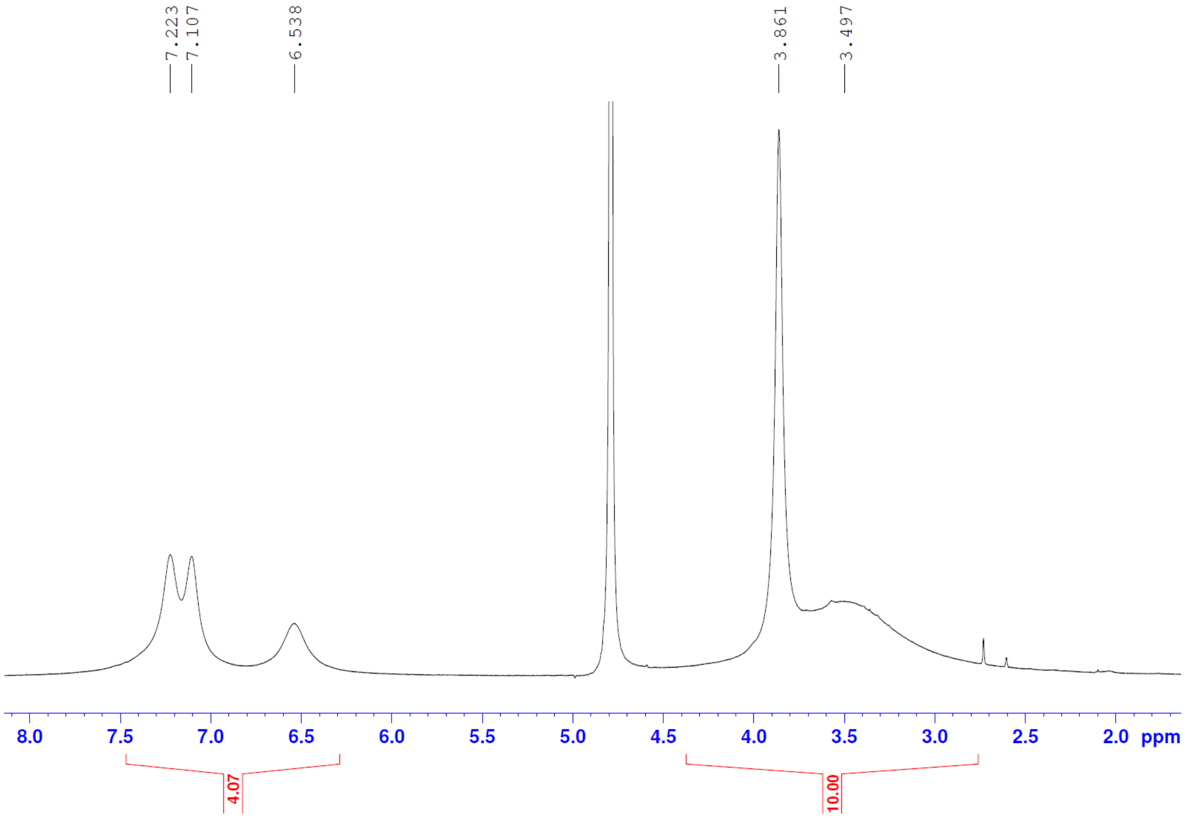

Figure S1. ^{1}H-NMR spectrum of celestine blue (CB) in D_2O at room temperature

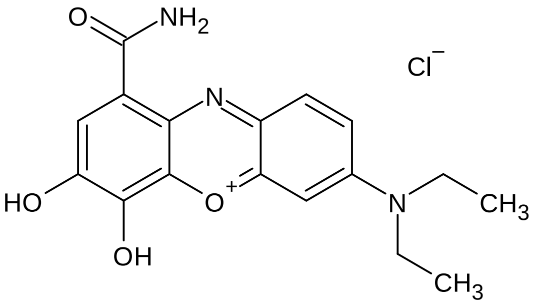

Figure S2. The molecular structure of celestine blue (CB)

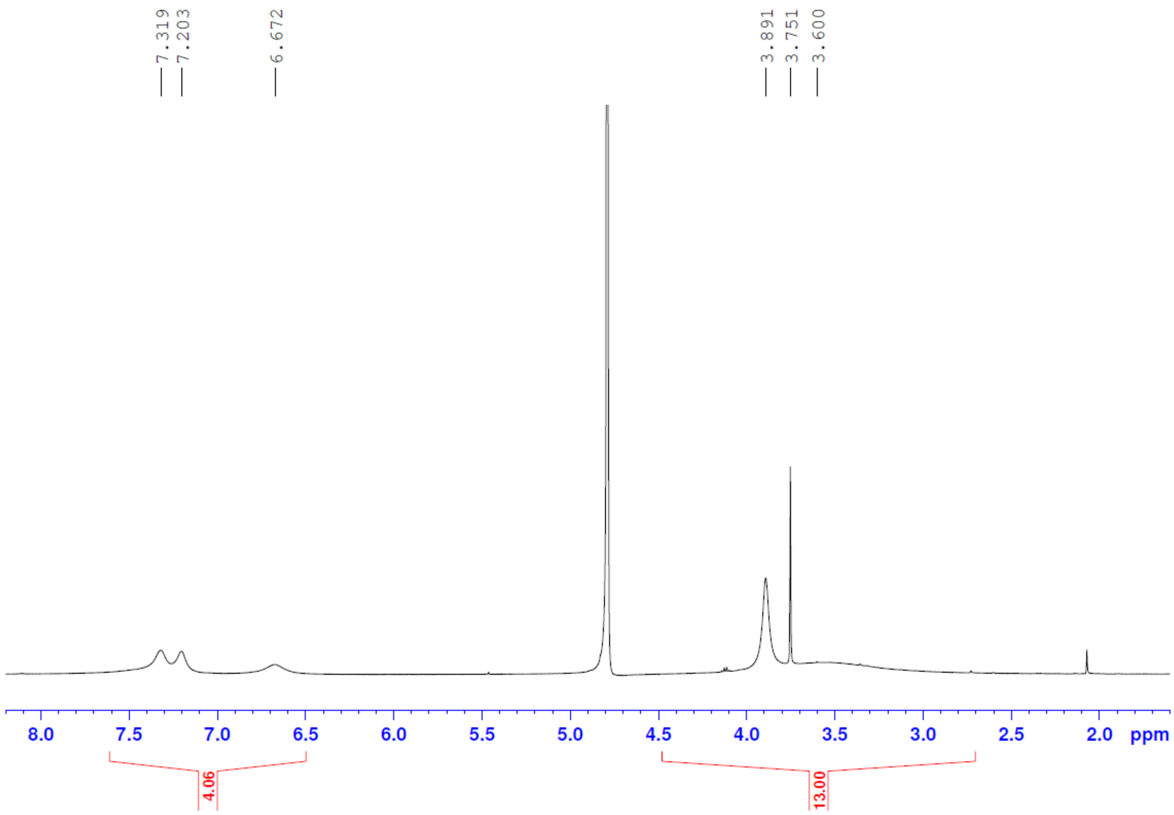

Figure S3. ^{1}H-NMR spectrum of methyl celestine blue (mCB) in D_2O at room temperature

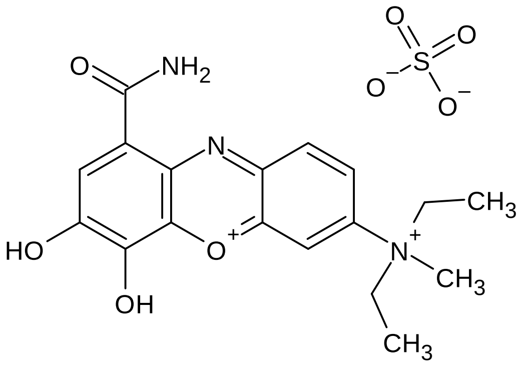

Figure S4. The molecular structure of methyl celestine blue (mCB)

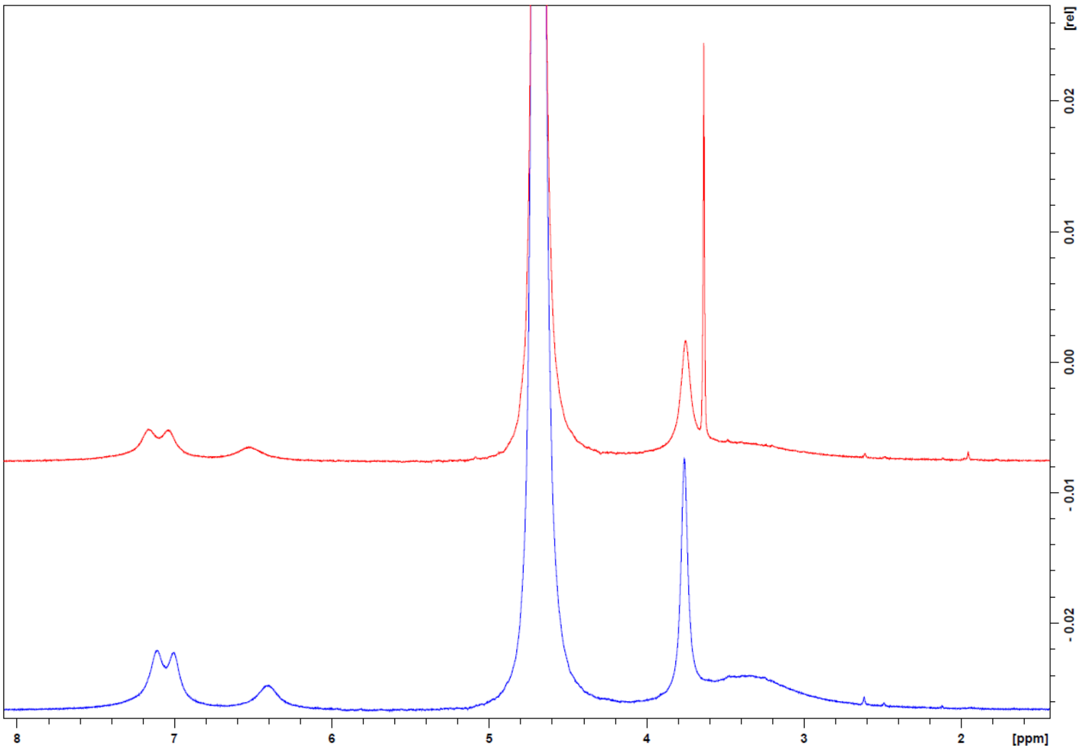

Figure S5. The ^{1}H-NMR spectra comparison of celestine blue (blue) and methyl celestine blue (red) in D_2O at room temperature

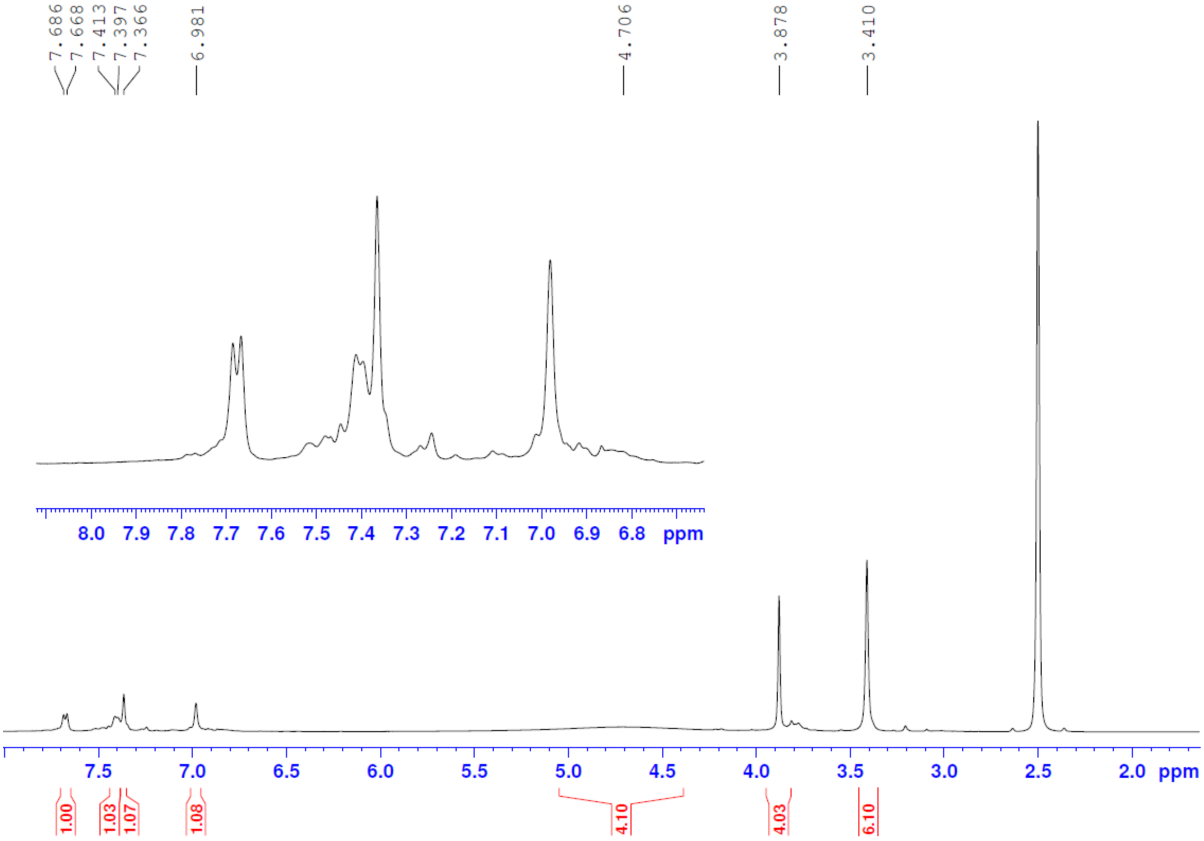

Figure S6. ^{1}H-NMR spectrum of celestine blue (CB) in d_6-DMSO at room temperature

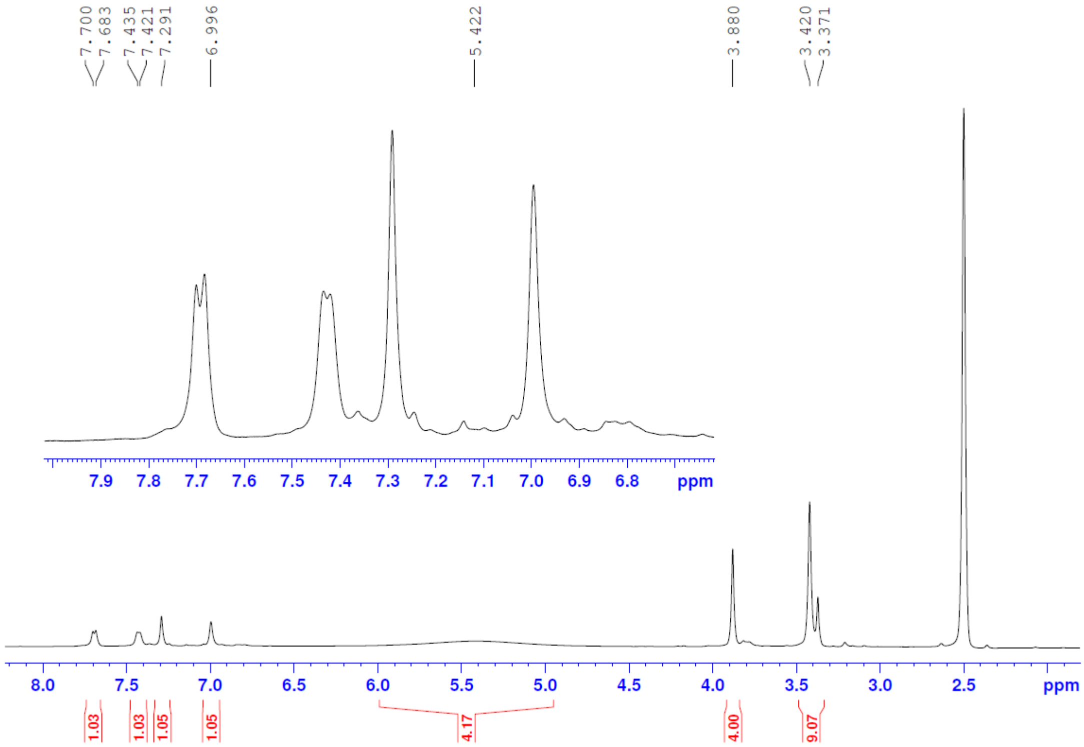

Figure S7. ^{1}H-NMR spectrum of methyl celestine blue (mCB) in d_6-DMSO at room temperature

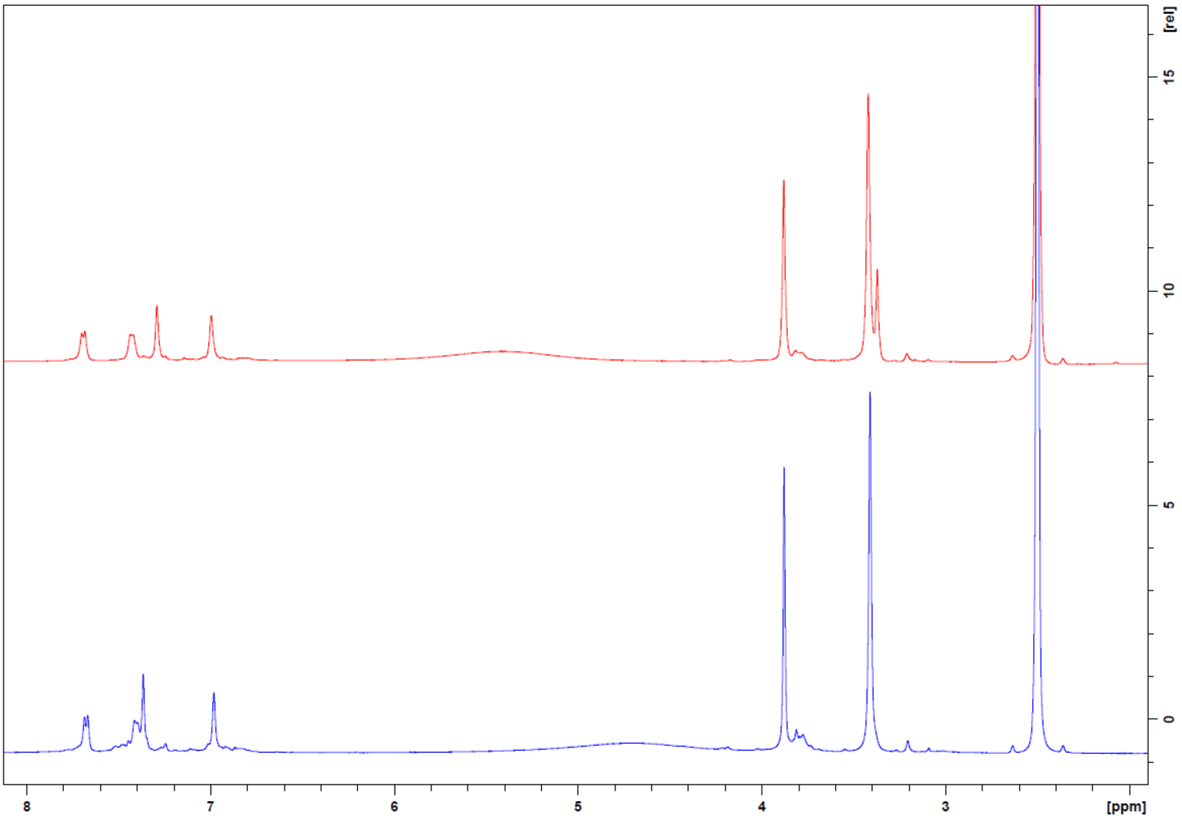

Figure S8. The ^{1}H-NMR spectra comparison of celestine blue (blue) and methyl celestine blue (red) in d_6-DMSO at room temperature

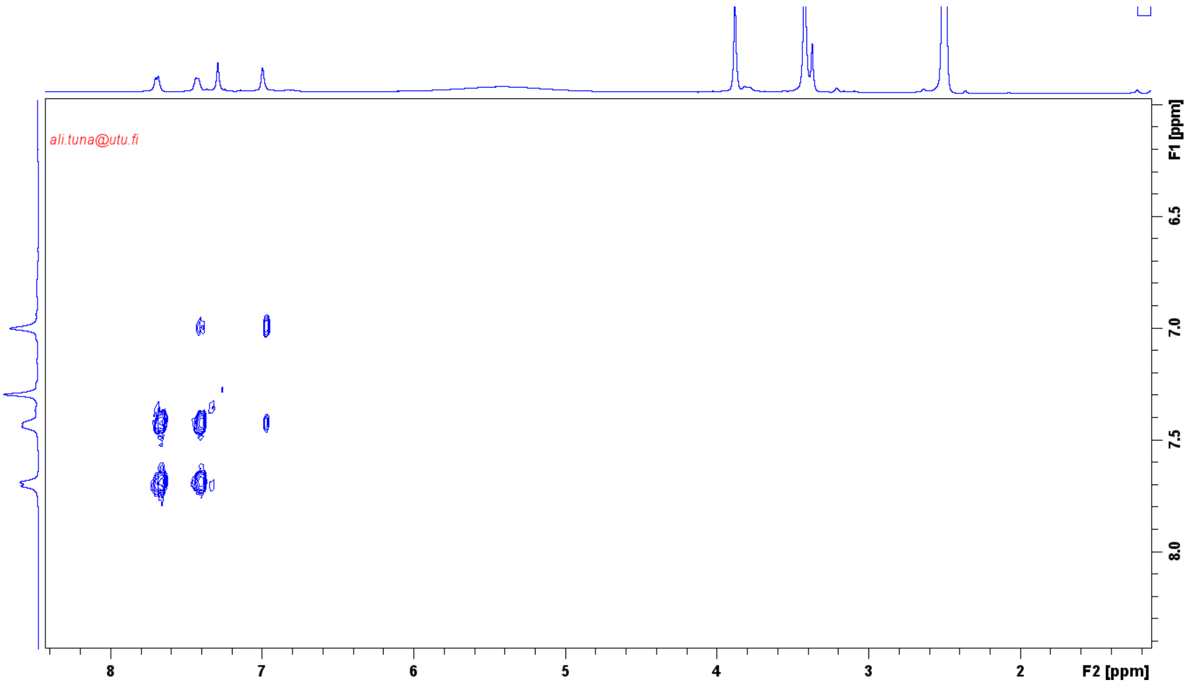

Figure S9. The 2D-COSY NMR spectrum of methyl celestine blue in D_2O at room temperature

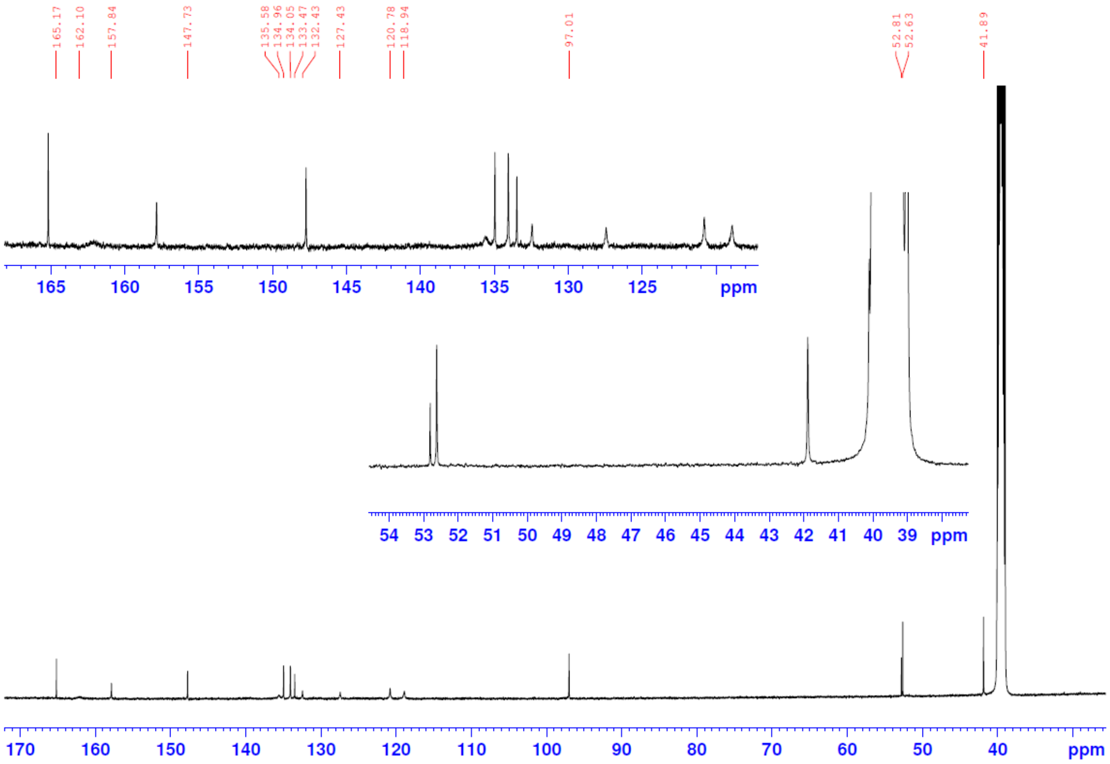

Figure S10. ^{13}C-NMR spectrum of methyl celestine blue (mCB) in d_6-DMSO at room temperature

Created by: oligo, oligo
Created on: Apr 25, 2024
Item name: Low Mass Positive Apr 25, 2024 10:17:44 FLE Daylight Time   Created time: 12:00:11 FLE Daylight Time

Item name: Blank

Item name: Blank, Sample position: 1:A,1, Replicate number: 1

.	Component name	Identification status	Neutral mass (Da)	Observed neutral mass (Da)	Observed m/z
1	mCB_AT.mol	Identified	343.15321	343.1501	382.1132

.	Component name	Identification status	Mass error (mDa)	Mass error (ppm)	Expected RT (min)	Observed RT (min)
1	mCB_AT.mol	Identified	-3.1	-8.2		0.66

.	Component name	Identification status	Detector counts	Response	Adducts
1	mCB_AT.mol	Identified	661	628	+K

Component name: mCB_AT.mol

Item name: Blank
Channel name: mCB_AT.mol [+K] : (68.2 PPM) 382.1132

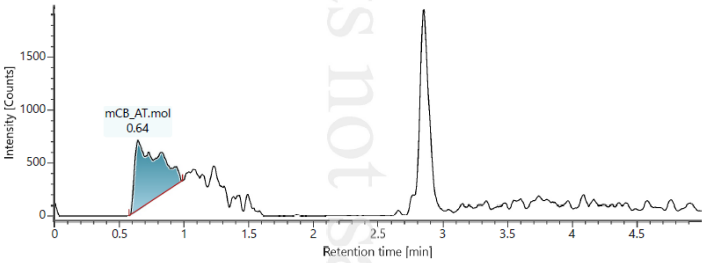

Figure S11. The HR-MS analysis report of methyl celestine blue (mCB) in water

Created by: oligo, oligo
Created on: Apr 25, 2024
Item name: Low Mass Positive Apr 25, 2024 10:17:44 FLE Daylight Time   Created time: 12:04:34 FLE Daylight Time

Item name: B 10 uM   Channel name: Low energy : Time 2.8608 +/- 0.0413 minutes
Item description:

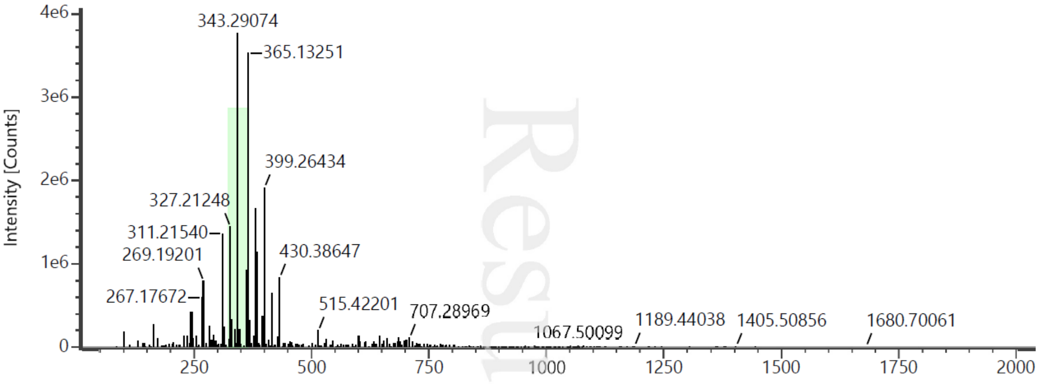

Item name: B 10 uM   Channel name: High energy : Time 2.8608 +/- 0.0413 minutes

Figure S12. The HR-MS spectrum of methyl celestine blue (mCB) in water

Solid and liquid FTIR-ATR analysis

The solid and liquid states FTIR-ATR measurements were performed using Bruker Vertex 70 FTIR-ATR spectrometer in solid and liquid states due to the stability of radical specie of reduced methyl celestine blue either in H_2O or D_2O–H_2O mixture. The obtained spectra were analyzed over Origin and Microsoft Excel (Figure 5 and Figure 6).

The solid state FTIR-ATR spectra were obtained on gold crystal and the resulting spectra were baseline-corrected versus air with 128 scans. The spectra were used for comparison of celestine blue and methyl celestine blue species. Upon methylation, some peaks were defined to be, slightly increased aliphatic C-H stretching, a strong C-H scrissoring (”methyl rock”), and C-H bending change due to aromaticity change at the wavelengths of 2800 cm-1, 1150 cm-1, 600 cm-1 respectively.

The liquid state FTIR-ATR spectra were also obtained on gold crystal and the resulting spectra were baseline-corrected versus water with 128 scans. The spectra were used for comparison of celestine blue, methyl celestine blue and reduced (radical) methyl celestine blue species. Around 3000 cm-1 slightly increased due to ring current which becomes more negative. around 2500 cm-1 a sharp peak due to radical behavior, around 1600 cm-1 the reversed stretching due to the changed aromaticity with a radical specie, around 1400 cm-1 an increased sharp peak due to C-H, ring current due to methylation but then disappeared while a radical formation which increase the ring current.

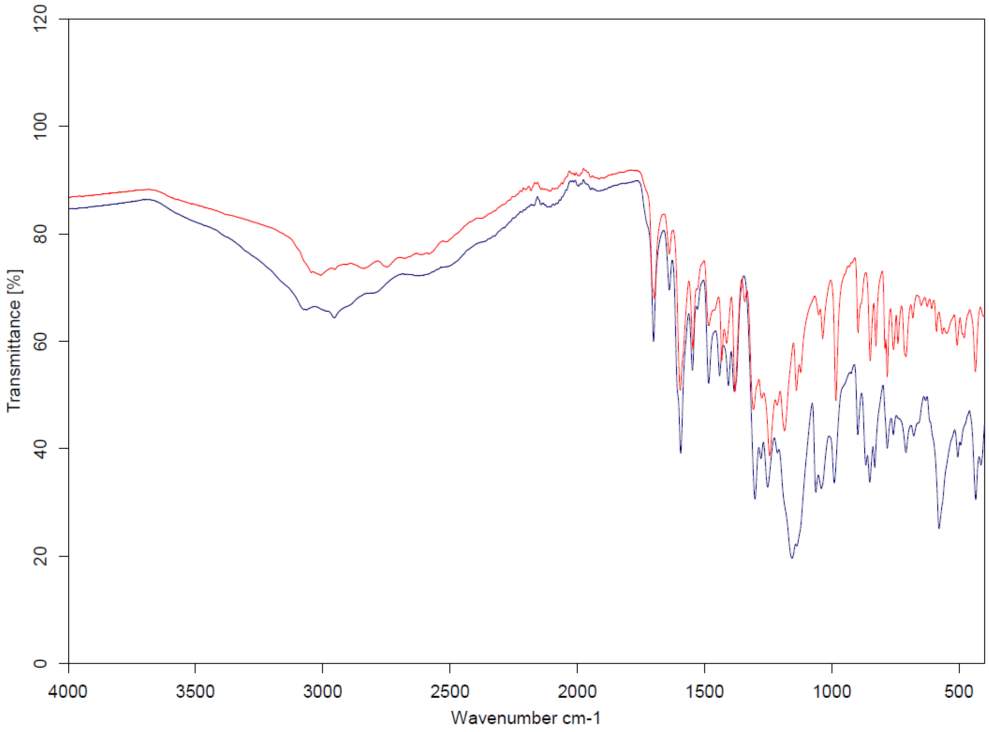

Figure S13. The solid-state ATR-FTIR spectra comparison of celestine blue (red line) and methyl celestine blue (blue line)

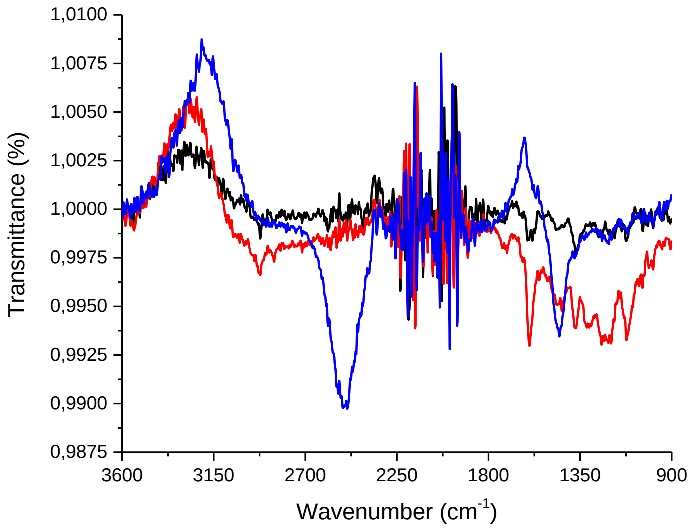

Figure S14. The liquid-state ATR-FTIR comparison of celestine blue (black line), methyl celestine blue (red line), and reduced methyl celestine blue (blue line) in H_2O or H_2O/D_2O

Electrochemical experiments

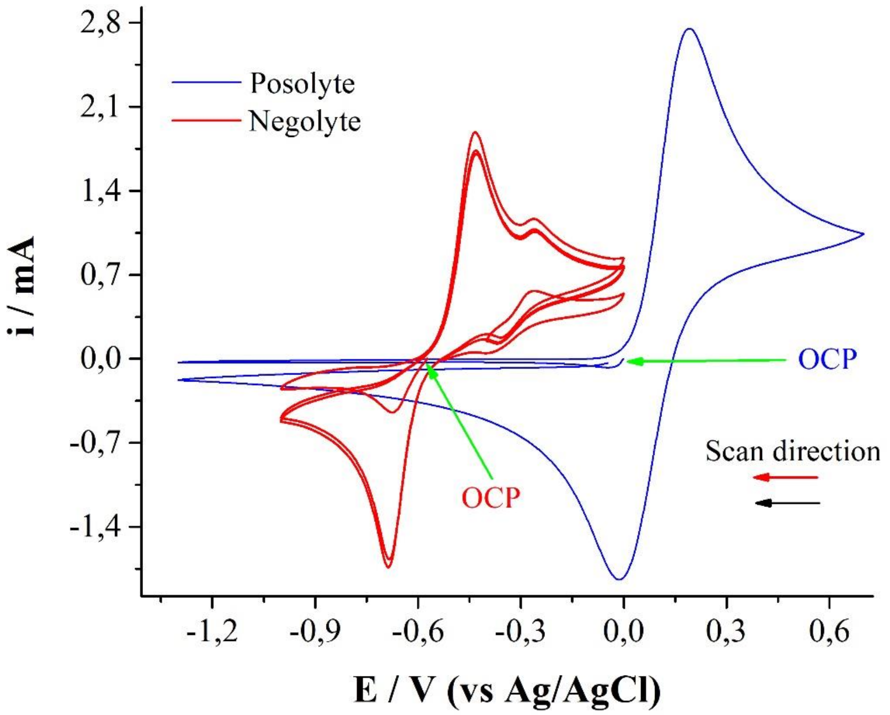

Figure S15. Cyclic voltammograms for posolyte and negolyte electrolytes recorded outside a glovebox at the end of the FB test, starting from OCP. Additional data on the solutions can be found in **Figure 5B** of the main text. Scan rate of 0.1 Vs^{-1} and GC electrode (d = 3mm). It can be observed that both solutions are predominantly reduced, with a new signal appearing at the negolyte solution. The signal indicates the presence of a byproduct. At least by cyclic voltammetry, no crossover of species is observed on the posolyte side.

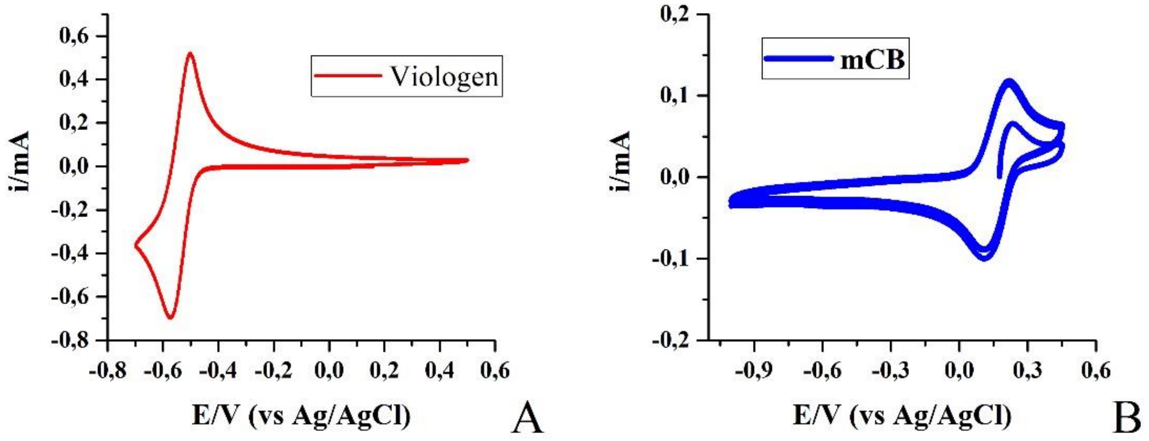

Figure S16. Cyclic voltammograms for posolyte and negolyte (pH changed from 7 to 4) electrolytes recorded at the end of the battery test. Additional data on the solutions can be found in **Figure 12** of the main text. Scan rate of 0.1 Vs^{-1} and GC electrode (d = 3mm). From the figure it can be seen that the viologen solution is discharged upon contact with air, while the **mCB** compound remains partially charged, exhibiting its air stability.

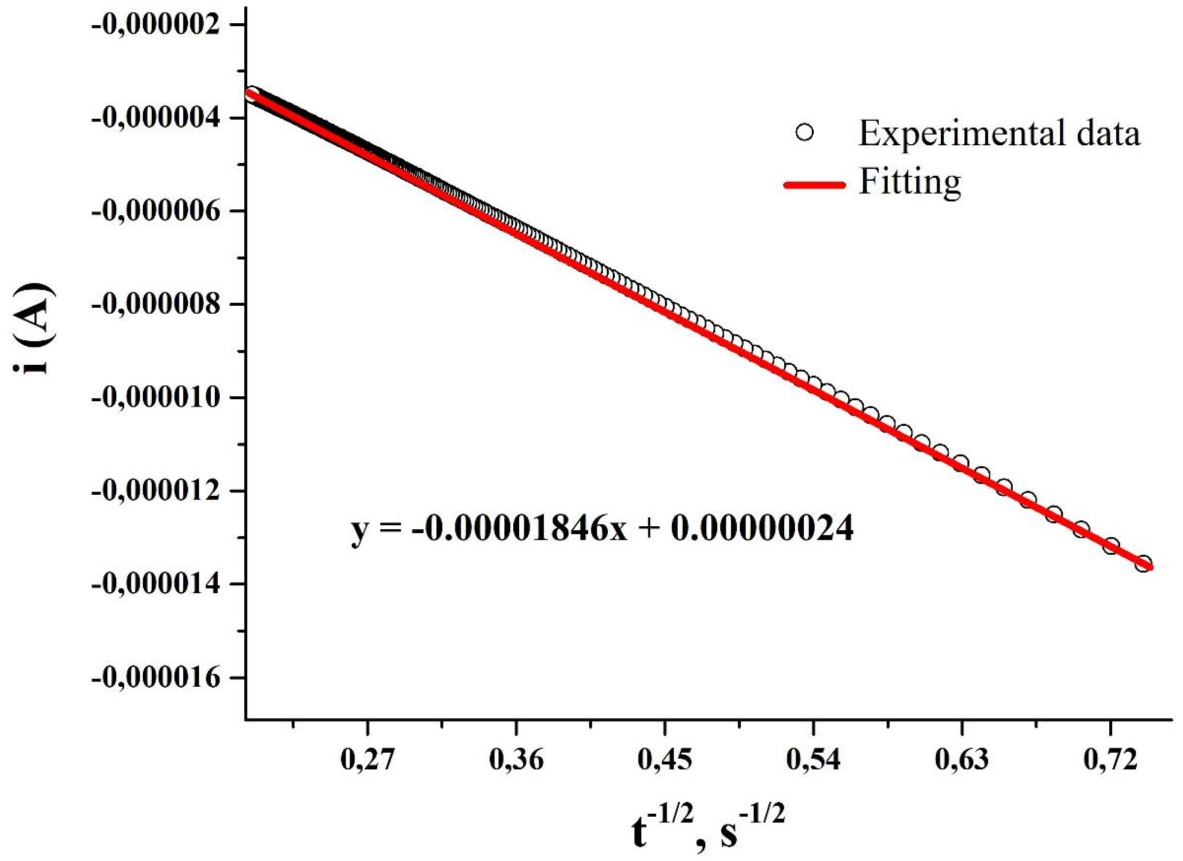

Figure S17. Estimation of the diffusion coefficient (Do) for the reduction processes of 2 mM **mCB** in 1 M KCl by Chronoamperometry. A GC disk (d=3 mm) was utilized in the application of 0 V (vs Ag/AgCl, 3M KCl) to the solution. Do, was estimated from Cottrell equation and resulted as Do = 5.76x10^{-6} cm^2 s^{-1} [1].

Voltammetric simulations:

To analyze the influence of the adsorption reaction at the electrode surface on the overall electron transfer process, cyclic voltammetry experiments were performed using a low analyte concentration (0.0025 molL^{-1}) and high scan rates (**Figure S15,** 5, 7.25 and 10 Vs^{-1}). As the scan rate increased, the cathodic signal began to broaden, and the reverse current grew significantly more than the reduction peak. This observation is in line with the occurrence of a mixed mechanism involving both outer- and inner-sphere electron transfer reactions [2]. Consequently, we used the DigiElch software 8FD to simulate the reactions described in equations S1 and S2 to estimate the apparent value of k_s, since the electron transfer process in reaction S1 is slower than in reaction S2. In other words, the electron transfer process taking place at the GC surface is faster than on the modified electrode (because its electronic properties changed). With this assumption, the value of $k2_s$ was supposed to be very large (ks2 = 100 cms^{-1}). As an initial approximation, α was set to 0.5, but α was also varied to more accurately reflect the shape of the voltammograms, as suggested by Waelder and Maldonado [3]. Considering the electrode area and scan rate values similar to those used in the real

experiment, simulated CV responses where obtained and presented in **Figure S15**. The main parameters controlling the processes were subtracted from simulations and compiled on **Table S1, Table S2** and **Table S3**.

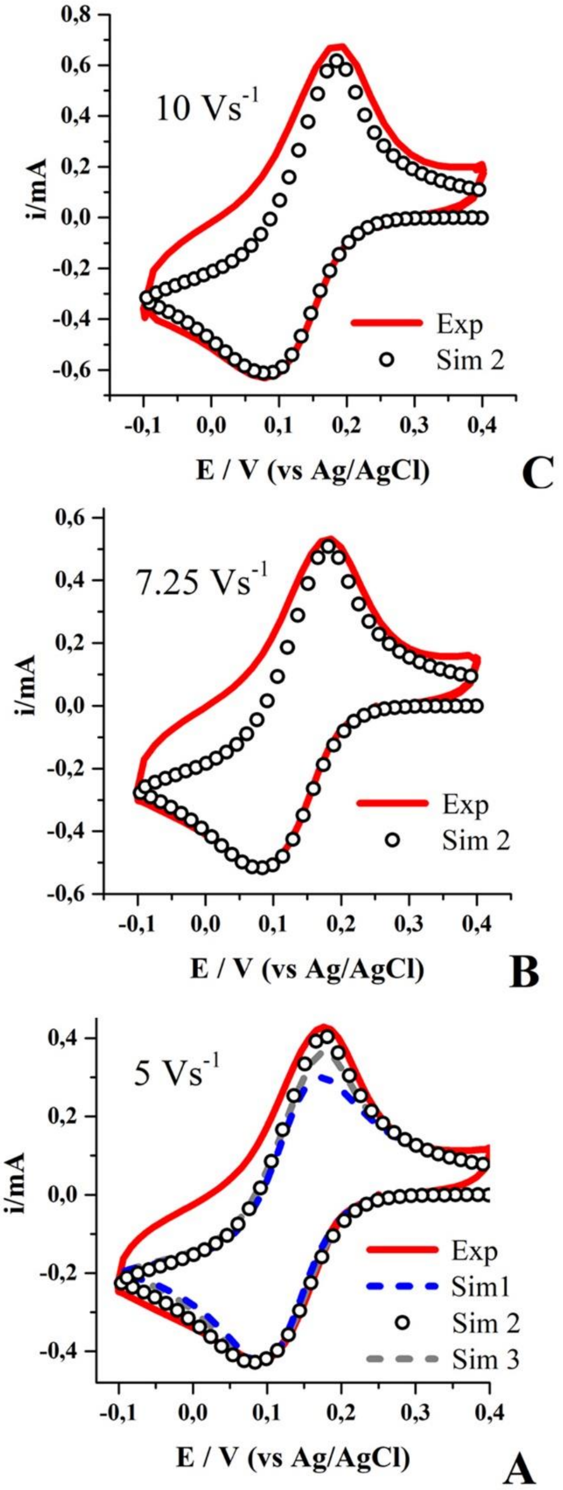

Figure S18. Simulated (sim) and experimental (Exp) cyclic voltammograms for the reduction process of 0.0025 molL^{-1} **mCB** at different scan rates with a GC electrode (d = 3mm). The simulations account for a simple electron transfer process (Sim 1, equation 4) and a mixed mechanism (described by equations 4 and 5) with α values of 0.4 (Sim 2) and 0.5 (sim 3).

Diffusion-controlled reactions occurring at the modified working electrode:

$$\mathbf{mCB}_{ox}^{2+} + e^{-} \rightleftharpoons \mathbf{mCB}_{red}^{+\bullet} \qquad E^0, k_s \qquad \text{(S1)}$$

The apparent diffusion coefficient for this reaction is represented as **Do,ap**

Inner-sphere electron transfer reactions occurring at the surface of the electrode:

$$\mathbf{mCB}_{ox(ads)}^{2+} + e^{-} \rightleftharpoons \mathbf{mCB}_{red(ads)}^{+\bullet} \qquad E2^0, k2_s \qquad \text{(S2)}$$

Table S1. Thermodynamic and kinetic information subtracted from the fittings of experimental and simulated CV responses, considering an electron transfer process described by equation 4 and α = 0.5.

**Electron transfer reactions**				
Ox + e- = Red	E0 = 0.133 V	α = 0.5	ks = 0.12 cms^{-1}	Do,ap = $1.8x10^{-5}$ $cms^{2}s^{-1}$

Table S2. Thermodynamic and kinetic information subtracted from the fittings of experimental and simulated CV responses, considering a mixed mechanism (equations 4 and 5) and α = 0.5.

**Electron transfer reactions**				
Ox + e- = Red	E0 = 0.125 V	α = 0.5	ks = 0.05 cms^{-1}	Do,ap = $1.65x10^{-5}$ $cms^{2}s^{-1}$
Ox* + e- = Red*	E* = 0.166 V	α* = 0.5	ks* = 100 cms^{-1}	

**Adsorption reaction**					
Ox=Ox*	K* $1.8x10^{5}$	kf* / $Lmol^{-1}$ $1.0x10^{25}$	a* 0.7		
Red=Red*	K* $9.0x10^{5}$	kf* $1.0x10^{25}$	a* 0	$τ_{max}$ $3.0x10^{-10}$	

Here, K* represents the equilibrium constant for the adsorption-desorption reaction of the oxidized and reduced species, while the adsorption rate constant is denoted by kf*. The software model assumes that, equilibrium, a Frumkin isotherm is recovered. A positive value of a* indicates that the molecules interact with each other at the electrode surface.

Table S3. Thermodynamic and kinetic information subtracted from the fittings of experimental and simulated CV responses, considering a mixed mechanism (equations 4 and 5) and α = 0.4.

**Electron transfer reactions**					
Ox + e- = Red		E0 = 0.125 V	α = 0.4	ks = 0.05 cms^{-1}	Do,ap = $1.95x10^{-5}$ $cms^{2}s^{-1}$
Ox* + e- = Red*		E* = 0.166 V	α* = 0.4	ks* = 100 cms^{-1}	
**Adsorption reaction**					
Ox=Ox*	K* $1.8x10^{5}$	kf* / $Lmol^{-1}$ $1.0x10^{25}$	a* 0.7		
Red=Red*	K* $9.0x10^{5}$	kf* $1.0x10^{25}$	a* 0		
				τ_{max} $3.0x10^{-10}$	

From simulations (**Figure S18**), it is evident that the best fitting is achieved when considering the mixed mechanism and α values of 0.4. This is supported by the observation of a broader reduction signal and a sharper peak as the scan rate increases, which aligns with the experimental results. By examining the results compiled in the Tables, it is clear that omitting the adsorptive interaction in the kinetic analysis of voltammograms (blue line, sim 1) leads to an overestimation of the ks value. As an important point to highlight is that, if a simple electron transfer reaction is considered (blue line, sim 1), the experimental fitting is poor even to reproduce the broadening of the reduction wave (blue line, sim 1). Note that the simulation could be improved to fully adjust the voltammograms, but this would add more variables to the simulation, since the parameter that controls the capacitive current of the system would also have to be modified, and more than one layer of adsorbed material should be considered as well.

REFEENCES:

[1]. A. J. Bard, L. R. Faulkner. Electrochemical Methods: Fundamentals and Applications;

Wiley: New York, 2001. This reference is cited as 29 in the main text.

[2]. N. Kurapati, P. Pathirathna, C. J. Ziegler, S. Amemiya. Adsorption and Electron-Transfer Mechanisms of Ferrocene Carboxylates and Sulfonates at Highly Oriented Pyrolytic Graphite. *ChemElectroChem*. 2019, 6, 5651-5660.

[3]. J. Waelder, S. Maldonado. Beyond the Laviron Method: A New Mathematical Treatment for Analyzing the Faradaic Current in Reversible, Quasi-Reversible, and Irreversible Cyclic Voltammetry of Adsorbed Redox Species. *Anal. Chem*. 2021, 93, 12672-12681.
